# Effect of Porous Substrate Topographies on Cell Dynamics: A Computational Study

**DOI:** 10.1021/acsbiomaterials.3c01008

**Published:** 2023-09-15

**Authors:** Alyse R. Gonthier, Elliot L. Botvinick, Anna Grosberg, Ali Mohraz

**Affiliations:** † Department of Materials Science & Engineering, University of California, Irvine, Irvine, California 92697, United States; ‡ Department of Biomedical Engineering, University of California, Irvine, Irvine, California 92697, United States; § Center for Complex Biological Systems, University of California, Irvine, Irvine, California 92697, United States; ∥ Beckman Laser Institute and Medical Clinic, University of California, Irvine, Irvine, California 92697, United States; ⊥ Department of Surgery,University of California, Irvine, Irvine, California 92697, United States; # Edwards Lifesciences Foundation Cardiovascular Innovation & Research Center, University of California, Irvine, Irvine, California 92697, United States; ○ Department of Chemical & Biomolecular Engineering, University of California, Irvine, Irvine, California 92697, United States; ◆ The NSF-Simons Center for Multiscale Cell Fate Research, University of California, Irvine, Irvine, California 92697, United States; ∇ Sue and Bill Gross Stem Cell Research Center, University of California, Irvine, Irvine, California 92697, United States

**Keywords:** membrane tension, negative Gaussian curvature, bijel, computational model, cell shape

## Abstract

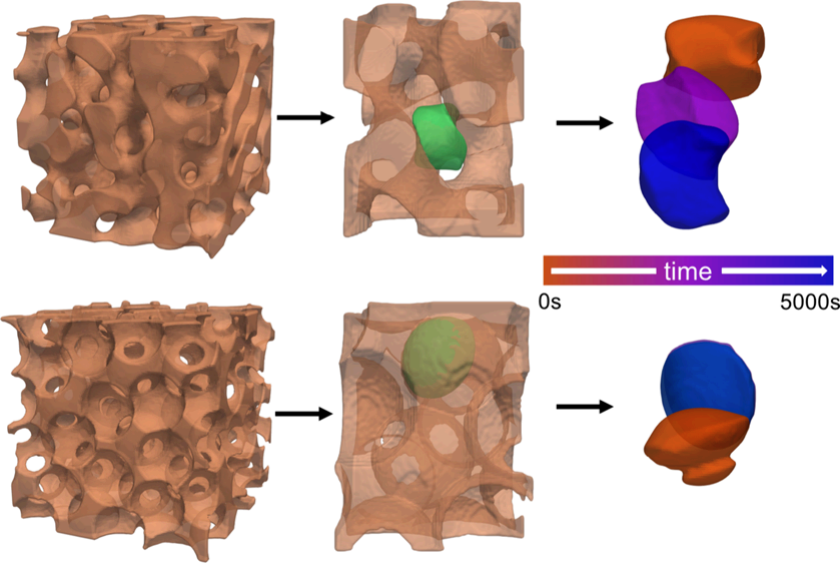

Controlling cell–substrate interactions via the microstructural characteristics of biomaterials offers an advantageous path for modulating cell dynamics, mechanosensing, and migration, as well as for designing immune-modulating implants, all without the drawbacks of chemical-based triggers. Specifically, recent *in vivo* studies have suggested that a porous implant’s microscale curvature landscape can significantly impact cell behavior and ultimately the immune response. To investigate such cell–substrate interactions, we utilized a 3D computational model incorporating the minimum necessary physics of cell migration and cell–substrate interactions needed to replicate known *in vitro* behaviors. This model specifically incorporates the effect of membrane tension, which was found to be necessary to replicate *in vitro* cell behavior on curved surfaces. Our simulated substrates represent two classes of porous materials recently used in implant studies, which have markedly different microscale curvature distributions and pore geometries. We found distinct differences between the overall migration behaviors, shapes, and actin polymerization dynamics of cells interacting with the two substrates. These differences were correlated to the shape energy of the cells as they interacted with the porous substrates, in effect interpreting substrate topography as an energetic landscape interrogated by cells. Our results demonstrate that microscale curvature directly influences cell shape and migration and, therefore, is likely to influence cell behavior. This supports further investigation of the relationship between the surface topography of implanted materials and the characteristic immune response, a complete understanding of which would broadly advance principles of biomaterial design.

## Introduction

Cell–substrate interactions are critically implicated in cell mechanosensing,^1−6^ migration,^7−9^ and morphology-induced behavior,^10−13^ leading to the utilization of this relationship in the development of functional biomaterials^14−24^ through both chemical and structural avenues. The impact of substrate nanotopography on cell behavior has been robustly tied to a mechanistic effect on cellular proteins of the same scale.^25−28^ However, such a relationship for microscale topography has not yet been fully established.^29−33^ Indeed, literature focusing specifically on the microstructure of implants suggests that control of the immune response is possible via biomaterial microscale morphology alone,^11,23,34−37^ which would eliminate any potential drawbacks to the introduction of chemical-based triggers.^38,39^

**Figure 1:**
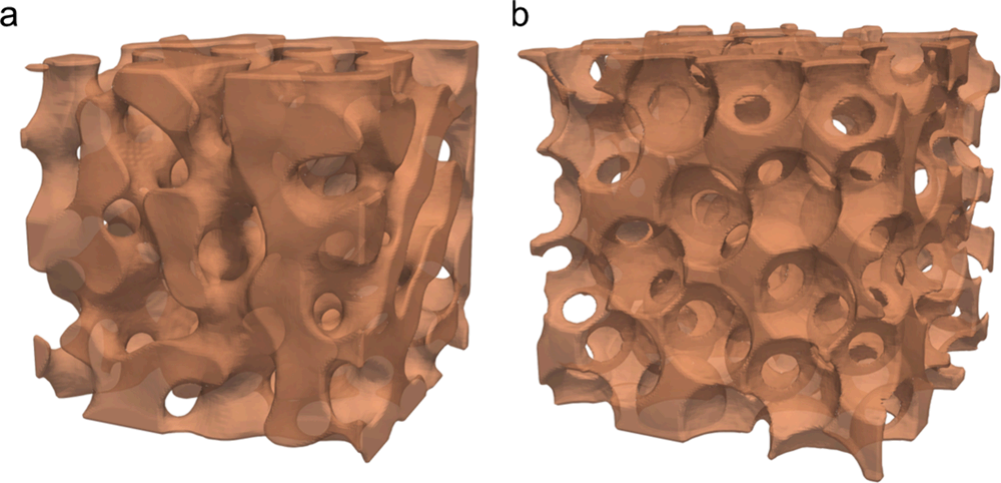
Representative computational substrates of a BTM (a) and a PTM (b) of approximately matching maximum pore widths.

To that end, a new class of porous material with unique microstructure, called a bicontinuous interfacially jammed emulsion gel (bijel)-templated material (BTM), was recently investigated as a potential immune-modulating implant.^20^ This material is derived from a bijel,^40,41^ resulting in a material with a continuous pore phase, negative Gaussian (saddle-like) curvature^42^ on all internal surfaces, and a uniform pore size throughout its volume (Figure 1a). These microstructural features arise due to the process by which bijels are formed:^43−45^ spontaneous demixing of two fluids by spinodal decomposition with neutrally wetting particles at their interface. By controlling the volume fraction of particles, the characteristic domain size of the resultant bijel can be prescribed.^46^ Once a bijel is formed, a photopolymerizable monomer solution, chosen to selectively partition into only one of the fluid phases, can be added and polymerized. The result of this process, the BTM, retains the unique surface curvature and pore size uniformity of its parent bijel and can be further processed for a variety of applications including electrochemical devices and regenerative biomaterials.^20,47^ In a subcutaneous implant study in rodents, the BTM demonstrated a favorable immune response.^20^ This favorability was classified by several factors, most notably the macrophage phenotype ratio (M2/M1) and depth of vascularization within the implant. In that study, the BTM was compared to another novel biomaterial, called the particle-templated material, or PTM, which elicits a much different immune response overall. The PTM shares some commonalities with the BTM, but it does not have the uniform pore size or negative Gaussian curvatures that are specific to spinodal structures such as BTMs. The PTM is formed by random close packing of polymeric microspheres, which are then brought past their glass transition temperature to allow the spheres to fuse slightly with one another, creating a single connected structure.^18,36,48,49^ The structure is subsequently inverted via the addition of a monomer solution around the fused microspheres, followed by photopolymerization and dissolution of the original particles, forming a porous structure with interconnected spherical pockets (Figure 1b). However, the pore arrangement in this structure is nonuniform, with pore sizes ranging from the original sphere diameter to the diameter of the connection point between adjacent spheres, referred to as an interconnect, which are typically only 30–35% of the sphere diameter.^18,36,49,50^ When comparing the BTM and PTM directly in the rodent study, the implanted materials were made from the same polymer and the pore size of the BTM was matched to the size of the PTM sphere diameter. Compared to the subcutaneously implanted PTM, the BTM yielded a notably larger percentage of M2 pro-healing macrophages relative to the total, as well as deeper vascularization within the porous implant after 2 weeks.^20^ All of the material properties except the landscapes of the surface curvatures were the same between the two materials. Therefore, it is prudent to investigate if the discrepancy between the M2 macrophage percentages of the two materials is related to the microscale curvature landscapes.

Immune cells involved in wound healing, such as macrophages, are well documented to modulate their phenotype and switch between pro-inflammatory (M1) and pro-healing (M2) types based on external physical cues.^10,11,37,51−54^ For example, macrophages that are more elongated tend to be predominantly M2 polarized,^52^ a cell shape driven effect. Other cell types also experience phenotype changes based on physical factors, such as the fibroblast-to-myofibroblast transition which is known to be affected by external mechanical cues, including curvature,^55,56^ as well as the connection between keratinocyte shape and differentiation.^57^ Many of the mechanisms directly connecting cell phenotypes and mechanosensing are still being investigated, though there is some evidence that this is related to the Rho/ROCK signaling pathway.^55,58−61^ Furthermore, some studies relate the effect of morphology on cell behavior distinctly to substrate curvature,^28,56,62−69^ which suggests the possible biological mechanism that differentiates cell behavior in the BTM versus the PTM.

To better understand the origins of this behavior and exploit it in the design of implantable devices, there is a need to rapidly and directly probe how cells respond to curved surfaces in porous materials. To this end, representative computational models that can replicate and systematically vary the salient features of these systems can be utilized to simulate the basic cell–substrate interactions at play in a simplified parameter space. This way, we can both understand the role of microstructural cues in mediating cell behavior on a broad level and inform decisions for future *in vitro* and *in vivo* experiments rapidly and with improved specificity. Initial efforts toward this goal are presented henceforth.

There are many existing computational models of cell behavior,^70^ with emphases from collective cell migration^7,71^ to ECM–lamellipodia interactions.^72−74^ Ideal models for probing the effect of microscale substrate curvature on cell shape and motion must incorporate the basic physics of cell migration and cell–substrate interactions with sufficient depth to replicate general behavior while maintaining computational practicality. In recent years, the use of the phase-field model has emerged as a computationally efficient treatment of cell migration as a moving boundary problem. Using one variable defined across the entire computational space, this type of model employs partial differential equations, derived from energy functionals, that define the evolution of phase-field variables and in turn enable tracking of cell shape, cytoskeletal behavior, and migration.^74,75^ One such model has utilized the phase-field approach in both 2D and 3D to approximate cell behavior,^76,77^ incorporating as few governing aspects of cytoskeletal organization as possible while maintaining the replication of gross *in vitro* cell shape and migration attributes. This model includes frontal-towing actin organization dynamics, cell–substrate adhesion, myosin contractility, and the relative tendency of the cell to conserve its volume, all initially calibrated via previous *in vitro* studies.^78^ In its 3D representation, this model accurately predicts movement along and confined by cylindrical channels, migration in vertically confined spaces, and alignment on micron-scale wave-patterned substrates.^77^ Notable exclusions from this model include focal adhesion attachment and organization as well as nuclear volume and deformation. While these exclusions are a distinct limitation of this type of model, the *in vitro* replication ability in response to substrate topography is nonetheless noteworthy and especially useful for examining complex substrates (e.g., Figure 1).

However, these existing models are not without limitations, which constrain their predictive power. Previous *in vitro* work has shown that cells generally tend not to rest atop the convex region of a curved (dome-like) surface of similar scale to a cell,^79^ thought to be related to stress fiber organization and unfavorable bending at the substrate contact region.^32,67^ In the previously published 3D model, cells that directly interact with comparable, convex dome-like surfaces do rest atop it and cease migration,^77^ which is nonphysiologic. Given that the new substrate of interest, the BTM, has a unique distribution of curved surfaces (negative Gaussian, zero mean curvature on all internal points), the 3D model in its existing form is not sufficiently equipped to accurately predict the cell behavior in this situation. The necessary modification to this model must therefore incorporate additional physics related to the influence of the substrate curvature. A separate 2D model has introduced a membrane tension term driven by changes to the surface area and local membrane curvature.^80^ In this paper, by adapting a similar term into a modified 3D model, we replicate the physiologic behavior of cells on the previously described convex curvatures (dome-like surfaces). Employing this new model, the effect of microscale substrate curvature on cell behavior in porous materials, namely, the PTM and BTM (Figure 1), is then examined. Our results suggest that BTMs and PTMs induce markedly different cell shape and migration profiles, contributing to the understanding of how microscale curvature affects basic cell behavior and providing direction for the investigation of how substrate curvature is related to vascularization and macrophage polarization within porous implants.

## Materials and Methods

### Model Development

The parameters of the phase-field model utilized in this study are summarized in Table 1. The model framework has been adapted from previous literature reports^76,77^ but is briefly summarized here. This nondimensionalized model describes the interaction of a generic cell parametrized by ρ and *p⃗*, which represent cell location and relative actin organization, respectively, through eqs 1 and 2 below. In this nonconserved phase-field model, the cell is defined by the region where ρ ≥ 0.5. For more control and consistency, we introduce a continuous definition of the membrane region, defined by a function that is 1 at ρ = 0.5 and decays to zero on either side, namely, 1-tanh^2^(*G*(ρ – 0.5)). The membrane thickness, and therefore the effective impact of the membrane tension term described below, can be tuned via *G*, which controls the steepness of the decay from ρ = 0.5. This generic cell interacts in 3D space with a substrate parametrized by Φ and Ψ, where Φ defines the substrate location and Ψ defines an interface that extends beyond Φ where an incoming cell can begin to detect that it is approaching an object. The evolution of ρ, which captures the cell dynamics, is defined by eq 1. The physical meaning of the terms are as follows: (*D*_ρ_∇^2^ρ) is a diffuse interface, (α∇ρ·*p⃗*) is the advection of ρ along *p⃗* that describes the relationship between the *p⃗*-field and the cell membrane, (κ∇Φ·∇ρ) is the adhesion of the cell to the substrate, (λρΦ^2^) is the exclusion of the cell from the substrate, and ((1 – ρ) (δ – ρ)ρ) is the motion of the cell boundary related to the cell volume. All constants and variables of eqs 1–3 are defined in Table 1. The parameter δ is specified in eq 3, and it describes, with strength μ, the tendency of the cell to conserve its initial volume. This parameter is also modulated by the inclusion of myosin motor contraction (σ|*p⃗*|^2^).
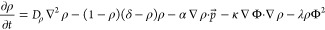
1
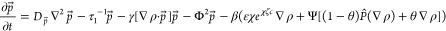
2
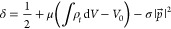
3

**Table 1:** Common Values for Model Constants Are Derived from the Ranges Present in the Existing 2D and 3D Models, Which Are Largely Based on Experimentally Determined Behavior^76,77,81^^a^

represented physics	symbol	value	represented physics	symbol	value
Diffusion of Cell Boundary	*D*_ρ_	1	Actin Generation	β	3
Diffusion of Substrate Boundary	*D*_Φ_	0.5	Diffusion of Substrate Sensing Boundary	*D*_Ψ_	4
Advection of the Cell with Actin	α	2	Weight of Membrane Tension	ε	0.34
Adhesion	κ	1–3	Acto-Myosin Contraction	σ	0.2
Exclusion of Cell from Substrate	λ	5	Symmetry Breaking of Myosin Motors at Cell Rear	γ	0.05
Diffusion of Actin Field	*D_p⃗_*	0.2	Ratio of Substrate Pushing Behavior	θ	0.4
Depolymerization of Actin	τ_1_^–1^	0.1	Strength of Volume Conservation	μ	0.001
Membrane Extensibility	*T*	1	Membrane Width	*G*	14

a Deviations from these values are explicitly noted. All values in this table are nondimensional. The Supporting Information contains detailed nondimensionalization of eqs 1–3, and Table S1 has example units for each of the parameters.

The overall strength and order of the actin field (*p⃗*) at a given location are defined by eq 2. The physical meaning of the terms are as follows: (*D*_*p⃗*_∇^2^*p⃗*) is the diffuse interface, (τ_1_^–1^*p⃗*) represents the actin depolymerization kinetics, (γ[∇ρ·*p⃗*]*p⃗*) is the asymmetrical distribution of myosin motor contractility force, (Φ^2^*p⃗*) is the exclusion of actin from the substrate, and lastly there is the actin source term with prefactor β. The actin source term described here deviates from previously published formats.^76,77^ In 2D, this term is simply given by β∇ρ, which describes actin polymerization at the cell membrane, oriented perpendicular to the interface.^76^ In 3D, this term takes the form Ψ[(1 – θ)*P̂*(∇ρ) + θ∇ρ], which introduces the tendency of actin to either push into a substrate perpendicularly (θ →1) or turn parallel to the substrate (θ →0); this term has previously been presented.^77^ The matrix operator *P̂* is therefore introduced to rotate the direction of actin polymerization for the latter case.^77^ The ratio of these two behaviors that a cell will exhibit is thought to be based on its specific phenotype or environment, although the parameter θ is not yet rigorously correlated to experimental values. In its original presentation and in this model, the value of theta (θ) is chosen from 0 < θ < 1, such that a given cell exhibits a ratio of pushing and nonpushing behavior. Due to the lack of rigorous experimental validation for this term, in this model, the value of θ was chosen within a range that maintained physiologic behavior, namely determined by the spreading of the cell. As this behavior exists only in proximity to the substrate, this term is also multiplied by Ψ.

In the adapted model presented here, the actin source term contains both the θ-containing term and an extrapolated membrane tension term, given by εχ*e*^χζ*c*^ ∇ρ, which was previously described in 2D form.^80^ The main component of this term ∇ρ(*e*^χζ*c*^) describes the exponential decay of actin polymerization with respect to forces exerted on the membrane (from both local curvature and changes in surface area),^82^ where *c* is the local curvature of the cell membrane given by  and ζ describes the change in surface area of the cell given by , with membrane compressibility modulus *T*, surface area *A*, and *f*_0_, which is a constant containing actin monomer size (*a*) divided by thermal energy (*k*_B_*T*).^80^ The reference surface area *A*_0_ is defined as the minimum surface area of the cell taken from a stationary, steady-state cell with no substrate interaction. The addition of the parameter χ mathematically specifies that this term only contributes to cell behavior at the membrane and is defined by 1 – tanh^2^(*G*(ρ – 0.5)) as described previously. In short, this term allows the modeled cell to “sense” high local membrane curvature and respond by slowing the actin polymerization in that direction. The coefficient ε weighs the effect of the membrane tension term with the original 3D θ-based term.

The exponential dependence of the membrane tension term on curvature is an approximation because the actual dependence of the formation of actin fibrils on the degree of curvature is likely cell phenotype dependent. As a result, it is important to show that the introduction of a curvature term impacts qualitative cell behavior in a stable manner, rather than to try to fit the exact quantitative location of the cell on a specific substrate (Figure S1).

The value of the ε coefficient was calibrated manually to determine the range at which the effect of the membrane tension term was significant, without allowing a nonphysiologic dominance of the actin source term (resulting in overspreading, for example). This way, the effect and significance of membrane tension can be effectively probed within the bounds of expected cell behavior.

Note that the original description of membrane tension in 2D also included a *D*_ρ_-modulating term in eq 1. Implementing this term in 3D yielded either nonphysiologic behavior, or such minimal significance that the effect was negligible; therefore, this term is omitted. An existing alternate adaptation of the membrane curvature in 3D also omits this term.^83^

The differential equations presented in eqs 1–3 were discretized and integrated numerically in MATLAB (ver. R2021b) by using central finite difference approximations to the derivatives. Most simulations were run for a total of 500 units of nondimensional time (corresponding to 5000 s), with some having longer runtimes if it was necessary for exploring long-term cell behavior. To probe for stability and convergence, the space was discretized to unit volumes of d*x* = d*y* = d*z* = 1, 0.5, and 0.25. The simulation is tightly convergent without the membrane tension term at any of these element sizes. However, the inclusion of curvature is known to be sensitive to spatial discretization.^84^ In the case of the phase-field model, this is further complicated by the need to define the lattice points that are associated with the membrane. As part of the fitting of parameters, it was confirmed that the same qualitative behavior of the results could be achieved if the membrane was manually defined for d*x* = 1 (as in previous implementations of this type of phase-field model^80^) and at *G* = 14 for d*x* = 0.5 and d*x* = 0.25 (Figure S1). As a result, we chose to complete all of the simulations with the more consistent hyperbolic tangent term and at d*x* = 0.5 (d*t* = 0.01) for more efficient runtimes. Time and spatial scales are set by τ_1_^–1^ and *D*_*p⃗*_ as 10 s and 1 μm, and eqs 1–3 are already nondimensionalized with respect to these scales; the nondimensionalization of each term can be found in the Supporting Information.^76,77,80^ The overall size of the simulation volume varied depending on the substrate, which was each initialized separately by importing or creating shapes natively in MATLAB and subsequently converting these to usable forms where the substrate exists initially at Φ = 1. The substrates are then relaxed for 3 or 5 time-steps to form the interaction interfaces for Φ or Ψ, respectively, via eqs 4 and 5.^77^ For consistency at different discretization scales, all the substrates were made and relaxed on a lattice of d*x* = 1 (corresponding to 1 μm) with d*t* = 0.05 and interpolated if necessary to be on the same lattice as the cell model.
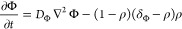
4
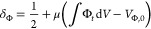
5

Cell size was approximated as an initial 17 × 17 × 17 cube (corresponding to 17 × 17 × 17 μm) for simplicity.^85^ Preliminary simulations were concurrently run with initially spherical cell shapes; however, no significant differences were observed between spherical and cube-shaped initial cells. The lack of sensitivity to initial shape was previously demonstrated.^77^ The simulations presented herein are spatially large enough that a migrating cell does not reach the outer bounds of the simulation volume, therefore eliminating any possible edge effects. For every individual case, the simulation volume extended beyond the combination of the substrate and initial cell position by at least 5 total spatial units (corresponding to 5 μm) in all directions. In all cases, the initial cell was placed without any substrate overlap, with an initial *p⃗*-field pointing straight down (*p⃗*_initial_ = −*e⃗*_*z*_, where *e⃗*_*z*_ is the unit vector in the *z*-direction) at every point within the cell, unless noted otherwise. To improve computational runtime, eqs 1–3 were solved for a given area, which includes the cell where ρ ≥ 0.01 and a “buffer” range of 1 μm beyond that value in every direction. No significant differences were found between simulations run within this buffer range and those run for the entirety of the simulation volume. The cell shapes defined by ρ = 0.5 were exported as STL files and visualized in Paraview open-source software. In the text, the “steady state” of a given cell–substrate system is defined to be past the point where any measurable cell movement or shape change ceases for more than 10 time-steps. All simulations are performed with the values listed in Table 1, unless otherwise stated.

### Substrate Development

A representative model of the 3D BTM substrate was formed by solving the Cahn–Hilliard phase-field equation,^86^ before being transferred to a binary matrix form in MATLAB. The Cahn–Hilliard equation is commonly used to simulate the evolution of a mixture undergoing spinodal decomposition, in the same way bijels are formed for the creation of BTMs. This process is characterized by the formation and subsequent coarsening of bicontinuous domains within the simulation volume as a representation of demixing by spinodal decomposition. The simulation was stopped when the domain size reached approximately 30–35 μm to best replicate the implantable structures used in previous *in vivo* studies.^20^

PTM substrates were formed directly in MATLAB, as randomly close-packed spheres of fixed radius (∼35 μm) with controlled overlap (∼30–35% of radius), as a representative case. This structure was built in consecutive layers to achieve a packing fraction of ∼0.64, corresponding to the random close packing limit of hard spheres, and was subsequently inverted by switching the solid and void phases within the structure.

## Fitting and Validation

**Figure 2:**
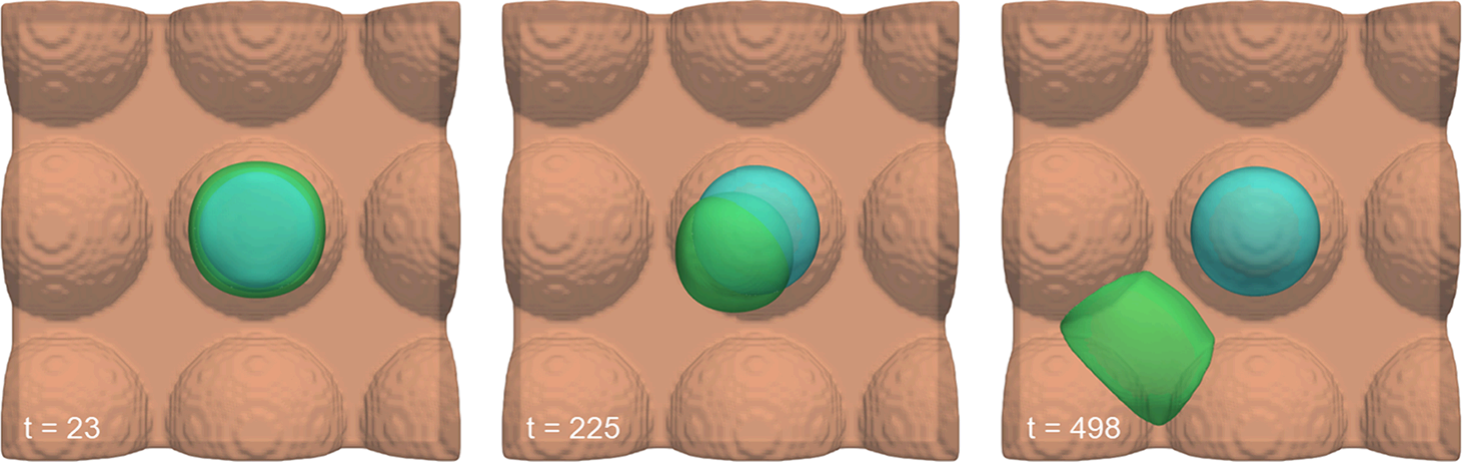
Evaluated separately and visualized at three progressive time points, a cell with membrane tension included (green) versus a cell excluding membrane tension (blue) was initialized moving toward a substrate with patterned hemispherical protrusions, designed to mimic *in vitro* data.^79^ Over time, the cell without membrane tension remained atop a hemispherical protrusion, whereas the cell with membrane tension replicated physiologic behavior, moving off the protrusion.

The model parameter ranges and values were validated using qualitative cell behavior in comparison to previously published *in vitro* data.^52,79^ Parameter ranges established in existing models were not refit.^76,77,80^ Initial validation was performed on a patterned surface of convex hemispheres, designed to resemble reported *in vitro* studies.^79^ This fitting and qualitative validation case was designed to demonstrate the importance of including membrane tension; the behaviors of cells with membrane tension present (green) and absent (blue) are compared in Figure 2. Both cells were initialized with identical initial conditions (in the same location directly above the approximate center of the full hemisphere and with the same initial *p⃗*-field). The cell with membrane tension moved down onto the hemisphere initially but then retracted slightly upward and moved laterally off the hemisphere and onto the flattened region. This end state is visually consistent with *in vitro* studies reported in literature.^67,79^ In contrast, the cell without membrane tension, effectively replicating the previous version of the model,^77^ remained directly atop the hemispherical protrusion. A full video of the case presented in Figure 2 can be seen in Video S1. Notably, this result is not sensitive to the lateral initial position; cells do not need to be initialized in a specific location above the hemisphere to achieve this result. There is a range of starting positions where a cell without membrane tension will still remain atop the hemisphere, while a cell with membrane tension continues to move off (Video S2).

This qualitative effect was achieved at all three discretizations (d*x* = d*y* = d*z* = 1, 0.5, or 0.25) and holds true for a variety of definitions of what is considered to be the membrane within the phase-field model (Figure S1). As such, we selected a combination of *G* and ε parameters in the middle of the range that reproduced the qualitative behavior matching experimental observations.

It is worth noting that, if the membrane tension term was alternatively chosen to be −β(εχ*e*^χζ*c*^∇ρ)(Ψ[(1 – θ)*P̂*(∇ρ) + θ∇ρ]), it is mathematically clear that the impact of membrane tension would be correlated to nonzero values of Ψ. This would imply that the effect of membrane tension, driven by local curvature and changes in surface area, is meaningful only at locations where the membrane is in contact with a surface, which is not a physiologically supported scenario. This argument therefore supports our choice to incorporate membrane tension by adding it independent of Ψ and the actin-turning term.

**Figure 3:**
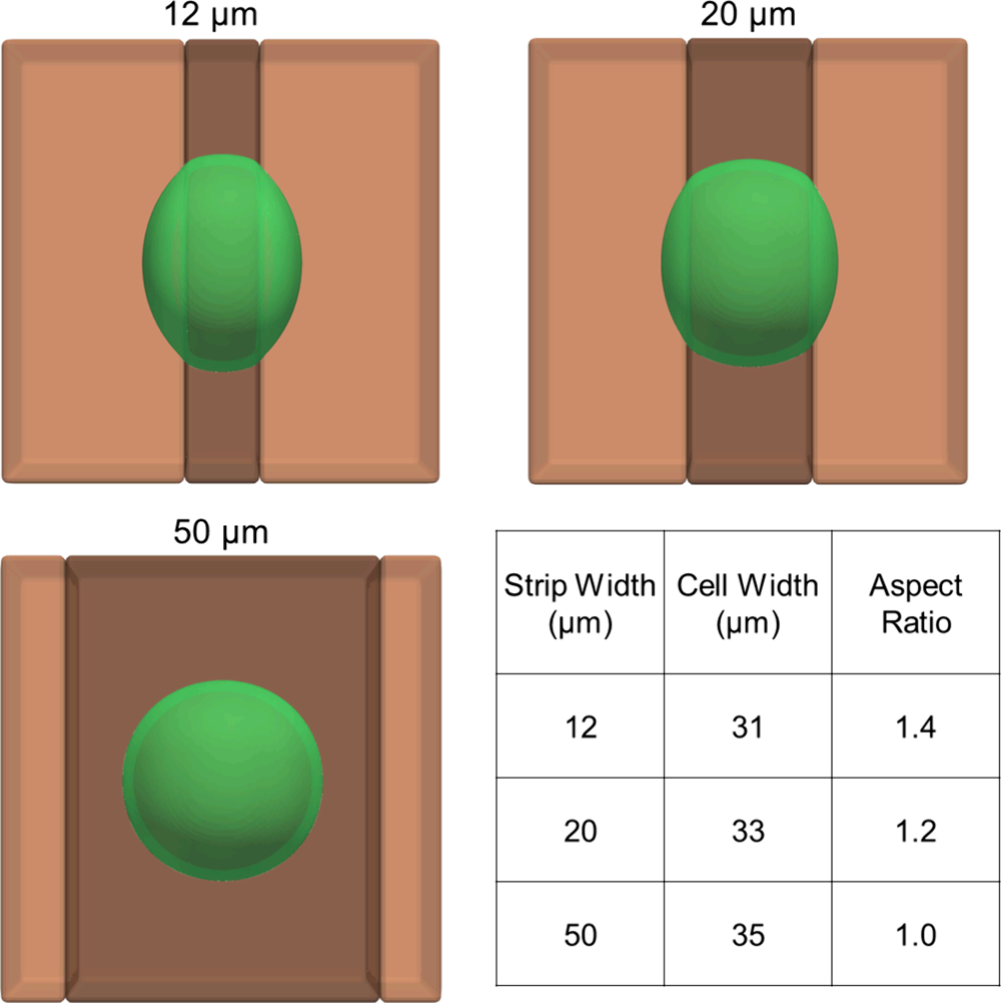
Cells interacting with flat substrates with centered adhesive strips of 12, 20, and 50 μm width, visualized at steady state. Quantification of cell width and aspect ratio in each case is summarized in the bottom right panel. The relative elongation of these cells grossly replicates existing *in vitro* behavior.^52^

One uniquely relevant *in vitro* study that connected cell shape to macrophage phenotype demonstrated that macrophages elongate on patterned adhesive strips of 20 μm width and preferentially polarize to the M2 pro-healing phenotype.^52^ To further quantitatively validate our model, we generated a similar substrate. Figure 3 shows modeled cells interacting with adhesive-strip surfaces, where the adhesive portion (κ = 5) is shown in dark brown and the nonadhesive regions (κ = 1) are light brown. The width of the adhesive strip is approximately 12, 20, or 50 μm, as noted in the figure. Identical cells were initiated above the patterned surface in the center of the adhesive strip in all cases. The images presented are shown in their final steady state. The aspect ratios of the modeled cells were calculated from the 2D projections of the cells onto the flat substrate. The relative elongation and lack of elongation of cells along the 20 and 50 μm strips, respectively, grossly replicate the change in aspect ratio demonstrated by macrophages *in vitro*.^52^ The 12 μm strip shown does not correlate to an existing *in vitro* experiment but instead demonstrates the potential utility of the model for predicting cell behavior under new conditions. This model does not account for lamellipodia or other cellular protrusions that are normally included in the *in vitro* calculation of aspect ratio, so experimental values will not match the model results exactly. For example, cells elongate to some extent on isotropic substrates *in vitro*. Thus, we emphasize that, most importantly, this model replicates the trend seen in the case presented.

## Results and Discussion

**Figure 4:**
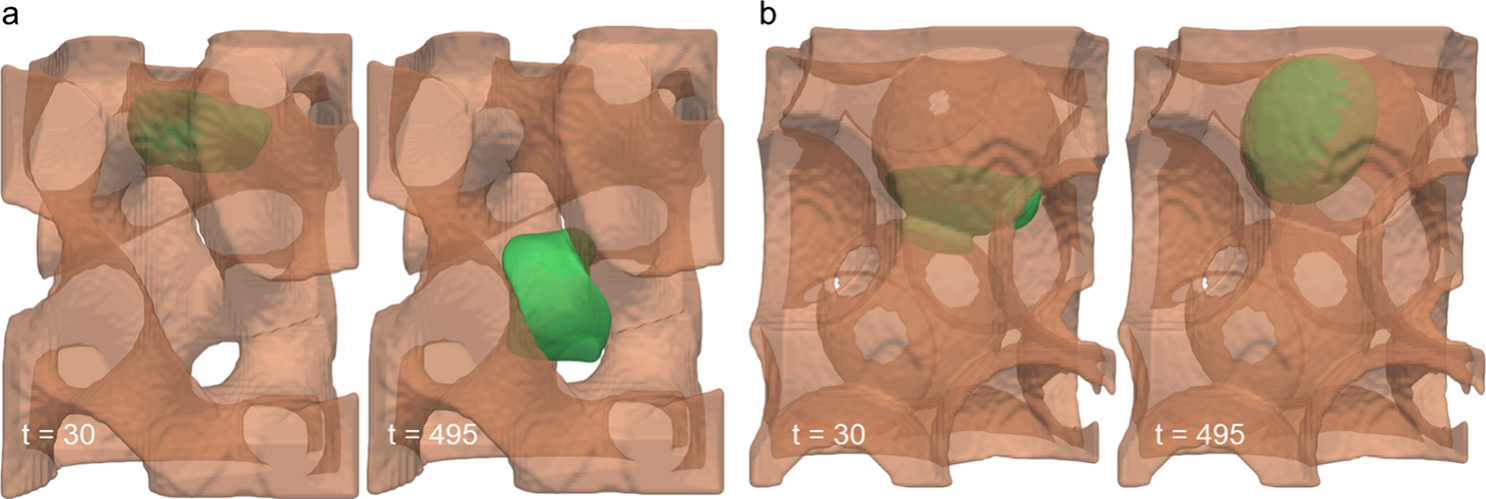
Cell migration through simulated materials, BTM (a) and PTM (b), at two time points. Substrates are displayed at 50% transparency for visualization purposes.

With the model sufficiently validated, we turned to investigating cell behavior on the uniquely curved internal surfaces of porous materials. For the BTM case, the cell was initialized above the substrate such that it would only contact the internal surfaces with negative Gaussian curvature, rather than the flat surface of the substrate’s outer edge. Once contact with the BTM was made, the cell migrated continuously through the porous structure for the entire simulation duration. The changes in shape and location of the cell within the BTM are presented at two representative time points in Figure 4a and in full in Video S3. On the PTM structure, the cell was originally placed in a pocket such that it would only contact its concave internal surfaces and not the flat outer portion of the substrate. In contrast to the BTM, the cell in the PTM interrogated one or more interconnects below its initial position and then retracted back into its original pocket, where it remained thereafter (Figure 4b). This was observed for all cases of cells interacting with the PTM structure (Figures S2 and S3).

**Figure 5:**
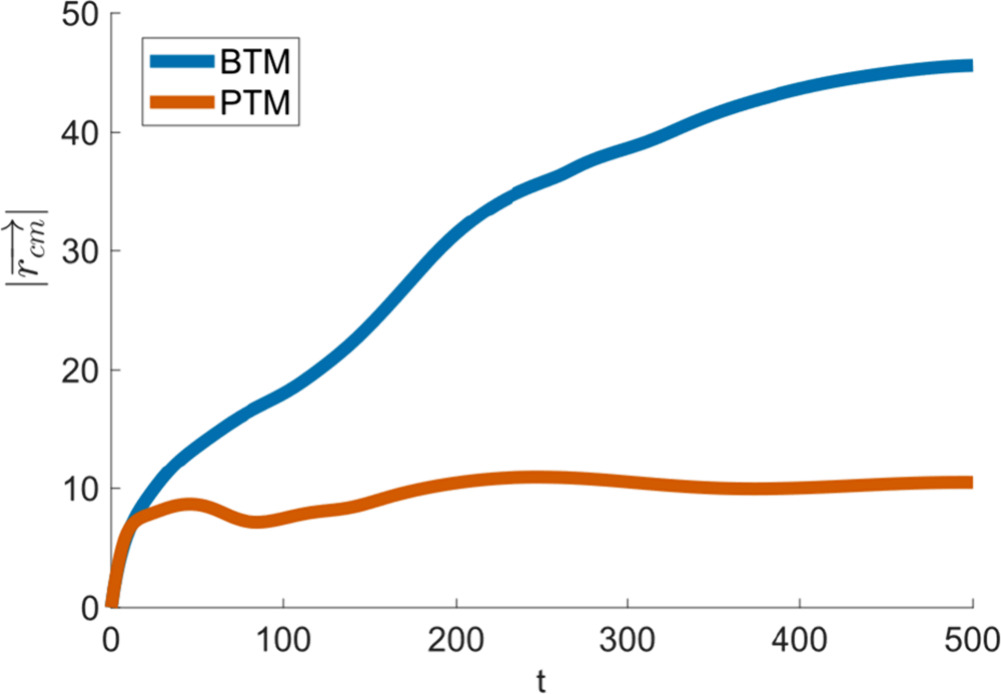
Displacement from the initial origin (*r*) of each cell is plotted over time, calculated via the centroids of the cell shapes formed by the ρ = 0.5 surface.

We first quantified the differences in behavior within the two porous structures by monitoring the distance traveled at each time point throughout the simulation, giving the displacement () from the initial position (Figure 5). This distance was calculated by computing the centroid of each cell shape formed by the ρ = 0.5 surface using the RigidBodyParams function^87^ in MATLAB and tracking the distance between the current and the initial centroid locations over time. Because an initial *p⃗*-field exists, an unhindered cell would continue to move away from the origin indefinitely, which would imply that  would continue to increase with time. The fact that it does not is a function of the interaction between the cell and the substrate. The cell velocity was also calculated as , using a centered difference approximation to the derivative. The calculated velocities, presented in Figure S4, confirm that the predicted cell dynamics are in proper physiologic range.^88,89^ The discrepancy in  between the two substrates is in agreement with existing *in vivo* data which shows deeper vascularization and collagen deposition into a BTM implant than into a PTM implant.^20^

**Figure 6:**
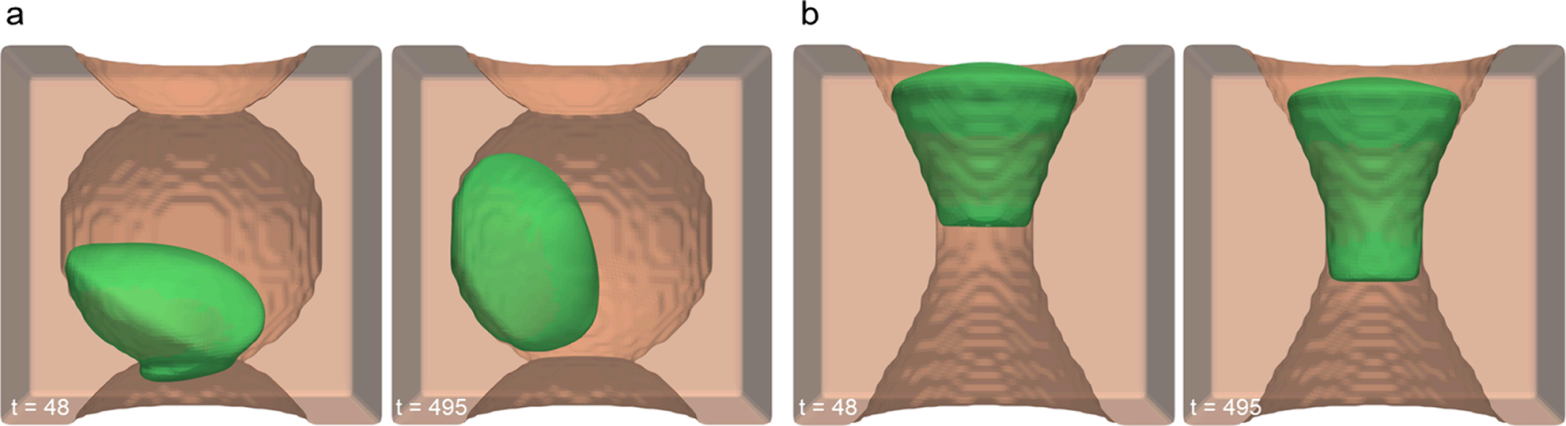
Origins of constrained motion in a PTM interconnect are further investigated via the differences between a standard PTM interconnect (a) and a hyperboloid (b) which has matching maximum and minimum diameters to the PTM interconnect.

Cell behavior within a PTM structure, according to this model, is limited to the outermost layers closest to the substrate boundaries, since all studied cases demonstrated that a cell will not cross the interconnect region linking two spherical pores. This type of trapping effect has previously been seen in other porous systems.^90^ In order to explicitly probe the interaction occurring between a cell and the interconnect, a simplified case of the PTM structure was created, henceforth termed the PTM interconnect. This simplified structure, visualized in Figure 6a, was designed as a vertical connection of three spheres of the same maximum pore and interconnect sizes as the original simulated PTM. For simplicity, data and simulations beyond this point investigate the cell dynamics within this simplified structure rather than the entire PTM itself.

The behavior of the cell in the PTM interconnect substrate (Figure 6a) is as follows: the cell was initially placed inside the middle spherical pocket with a prescribed *p⃗*-field of  and began moving down toward the lower interconnect. Upon interrogation of this high curvature region with the cell’s leading edge moving just through (*t* = 48 in Figure 6a), the cell slowed, stopped, and redirected to move in the opposite direction. The cell then briefly contacted the other interconnect above but again retreated into the original pocket and remained motile only inside the pocket for the remainder of the simulation (Video S4). Importantly, the cell did not settle with any part of it crossing the interconnect. To decouple the role of local substrate curvature from pore diameter in mediating this behavior, we performed simulations in a channel with the same maximum and minimum diameters as those in the PTM, but with different local curvature, utilizing the geometry of a hyperboloid (Figure 6b). The cell was initiated with the same conditions as the PTM interconnect case, placed such that it was above the smallest diameter region, as close as was geometrically allowed. This cell moved through the channel until a portion of the cell was past its narrowest region and then came to a stop, notably still inside the channel’s bottleneck (Video S5). By comparing the two cases presented in Figure 6, we show that the cell’s redirection when interacting with the PTM interconnect is not simply a matter of size exclusion and must instead be related to the local curvature and overall substrate landscape. While it is possible to increase the interconnect size, thereby lowering the local curvature at the pore throat, to the point that the cell passes through, this occurs at a pore overlap ratio far beyond what has been reported to be physically stable in the existing literature (Figure S5).

It is worth noting that the *p⃗*-field in all simulations throughout this work is a generic initial driving force of motion. The initial *p⃗*-field, as described in the Model Development section, was chosen to ensure physiologic velocity. Naturally, the cell behavior would change with different initial *p⃗*-fields. For example, with all else being the same, if the initial *p⃗*-field is very small, the cell migration would be very limited. In the future, the *p⃗*-field could be modulated by additional time-dependent inputs to represent the effect of the chemotaxis or other driving forces.

**Figure 7:**
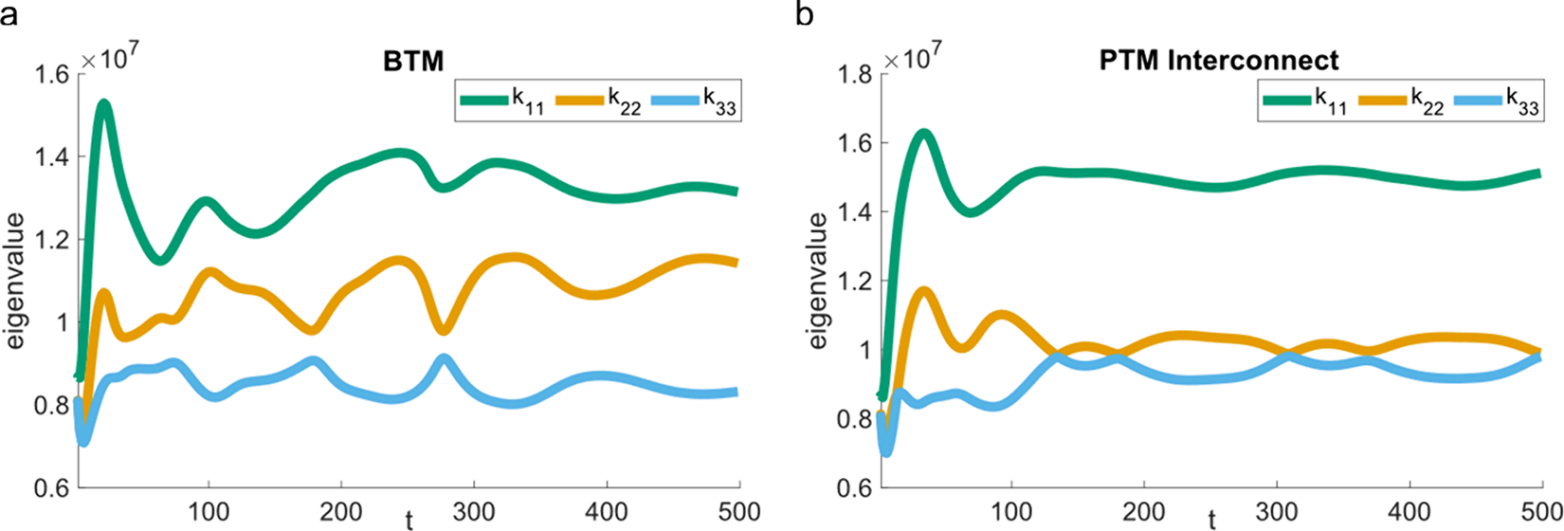
Eigenvalues of 3D cell shape on the BTM (a) and PTM interconnect (b), respectively, demonstrating the disk-like shape of the cell on the PTM interconnect in comparison to the BTM.

To quantify the differences between cells in the BTM and PTM interconnect substrates, we examined the general differences in cell shape due to its implication in cell behavior and specifically in macrophage polarization, as described previously. To achieve this, we quantified the overall cell shape via its eigenvalues. The eigenvalue matrix for a given 3D shape, diagonalized by reorienting the coordinate system to coincide with the shape’s principal axes, is represented by
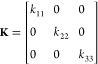
where the transformed eigenvalues *k*_11_, *k*_22_, and *k*_33_ quantify the relative scale of the shape along its three principal axes. The absolute values of *k*_11_, *k*_22_, and *k*_33_ scale with the size of the cell, while their relative values provide information about the shape in 3D. We used this general relationship to discuss the relative anisotropy of the cell shapes induced by the BTM and PTM interconnect. The eigenvalues were computed directly for the cell shape formed by the ρ = 0.5 surface using the RigidBodyParams function in MATLAB.^87^ In Figure 7, we monitor how these eigenvalues evolve for the two cells being studied. The cell in the PTM interconnect most closely resembles the eigenvalue relationship of a relatively flat, disk-like shape (*k*_33_ ≈ *k*_22_ < *k*_11_), consistent with our qualitative observations. This is in contrast to the cell within the BTM, which exhibits a comparatively more even spread of the three principal eigenvalues, suggesting a more complex shape without a distinct spherical or “flattened” nature. Our model also predicts a relationship between the cell–substrate contact area and the eigenvalues (Figure S6), suggesting that the cell shape is primarily governed by the curved surface with which the cell interacts.

To describe the behavioral differences of these cells and their relationship to the substrate landscape more holistically, we examined the energetic penalty associated with the shapes that a cell must adapt while migrating through a BTM or a PTM interconnect. In this context, we interpreted the topographies of the porous substrates as energy landscapes that the cell must navigate. To this end, we calculated a measure of cell energy solely due to its shape (*E*_shape_, eq 6) as a sum of the membrane energy from previously published work^80^ and the effective energetic reward from favorable interactions with the substrate. To account for the latter, we integrated the adhesion term from eq 1, following the Allen–Cahn formalism of phase-field models for nonconserved order parameters.^91^ We emphasize that this is only a measure of cell energy due to its shape and does not account for the energetics involved in actin polymerization or cell metabolism and function.
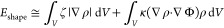
6

**Figure 8:**
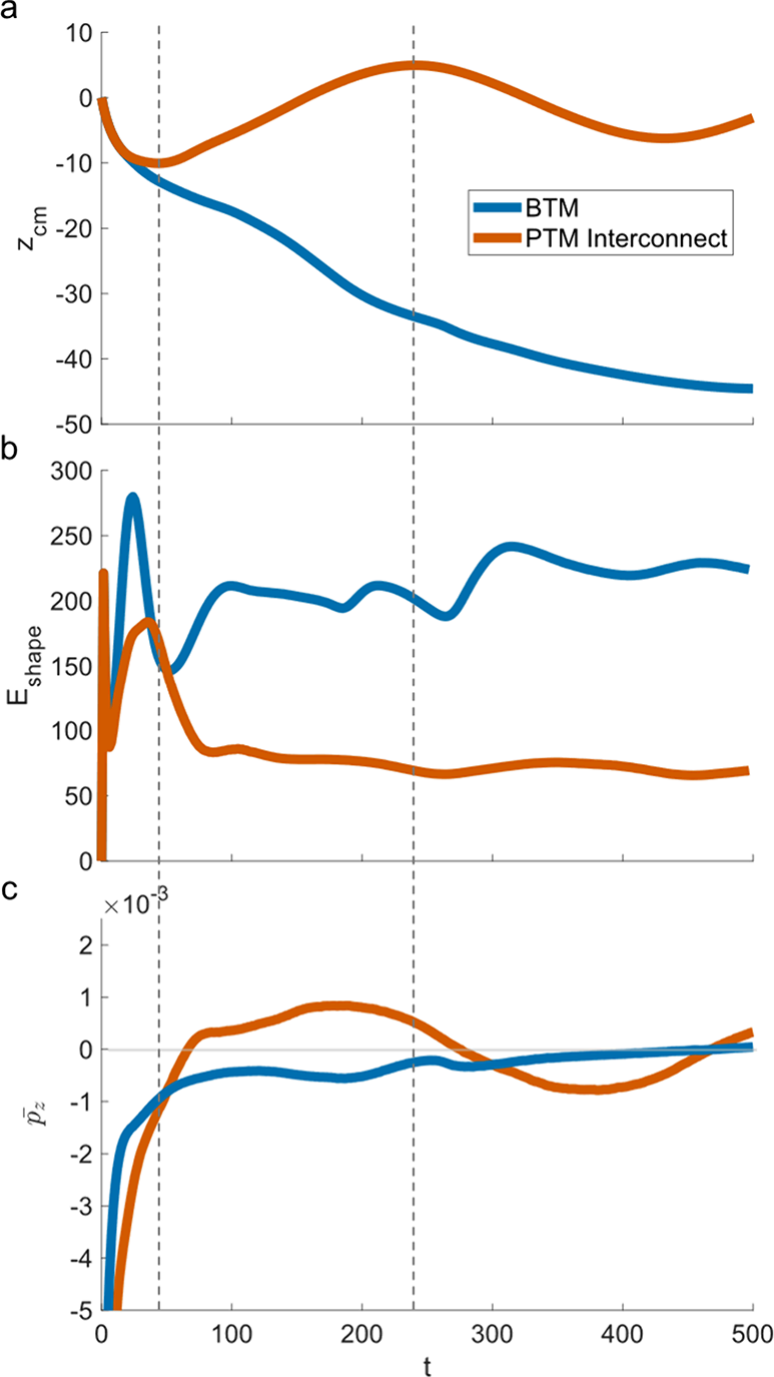
Changes in *z*-position from origin (a), shape energy (b), and mean *p⃗*_*z*_-field (c) are shown for the BTM and PTM interconnect cases. Time points of interest are marked by dotted lines at *t* = 45 (left) and *t* = 244 (right).

In Figure 8b, we compare the *E*_shape_ values for the two cells interacting with the BTM and the PTM interconnect, respectively. Note that the initial large peak in *E*_shape_ at *t* ≈ 23 seen in both cases is due to the initial contact with the substrates and will not be further analyzed. To calibrate our analysis of the changes in *E*_shape_, in Figure 8a, we monitor the *z*-component of the cell’s center of mass location, *z*_cm_, as well as the average *z*-component of the overall *p*-field, , in Figure 8c (recall the initial *p⃗*-field is pointing down, ). When viewed together, Figure 8a,b reveals important features that enable us to interpret cell migration through porous materials in the context of an energy minimization exercise. First, we denote the minimum value of *z*_cm_ for the PTM interconnect case (first dotted line at *t* = 45) after which the cell reverses its direction of motion following the interaction with the pore interconnect. Figure 8b shows that retracting back into the pocket allows the cell to lower its shape energy, as shown by the clear drop in *E*_shape_ after this point. In addition, Figure 8c shows a persistent sign reversal in  upon interaction with the pore interconnect until it reaches the interconnect at the opposite side, facilitated by the terms in eq 2, which is ultimately responsible for the cell reversing its migration course.

The second dotted line at *t* = 244 corresponds to the approximate instant when the cell reaches the pore interconnect on the opposite side (*z*_cm_ reaches its highest value). After this point, there is again a resultant sign reversal in . The shape energy does not meaningfully deviate from a small range of low energy states available within the confines of the pore, never again reaching through the interconnect as far as it does in the initial interaction driven by the initial *p⃗*-field (see Figure S7 for behavior up to *t* = 1000). We note that the peaks and valleys in *z*_cm_ or the instances of sign reversal in  do not always exactly coincide with peaks in *E*_shape_. This mismatch is not surprising as these parameters capture three separate measures of cell behavior. For example, small adjustments in cell shape after interaction with a high curvature region such as a pore throat can translate to changes in *E*_shape_ but may not necessarily impact *z*_cm_ in meaningful ways.

Similarly, a sign reversal in  may not immediately result in significant changes in the cell’s centroid position. Nevertheless, the close correspondences between the independent measures captured in Figure 8 enable us to explain the simulated cell dynamics in the context of an energy minimization exercise, mediated by how the local substrate topography may inform the actin polymerization dynamics within cells. In short, the most distinct behavior of the cell in the PTM interconnect occurs due to its interaction with the high curvature interconnect region, a high energy state that the cell in the PTM interconnect never reapproaches.

In contrast, *E*_shape_ takes a more consistently dynamic shape in the BTM due to its unique topography. A clear energy minimum state apparently does not exist in this case, causing the cell to continue its exploration through the porous material for the entire duration of the simulation. This correlates to the continuous change in *z*_cm_ for a cell on a BTM in Figure 8a, as well as a lack of redirection, such as that seen in the PTM interconnect case. This difference is also visible in Figure 8c, as the cell migrating through a BTM does not experience persistent sign reversals and large changes in .

**Figure 9:**
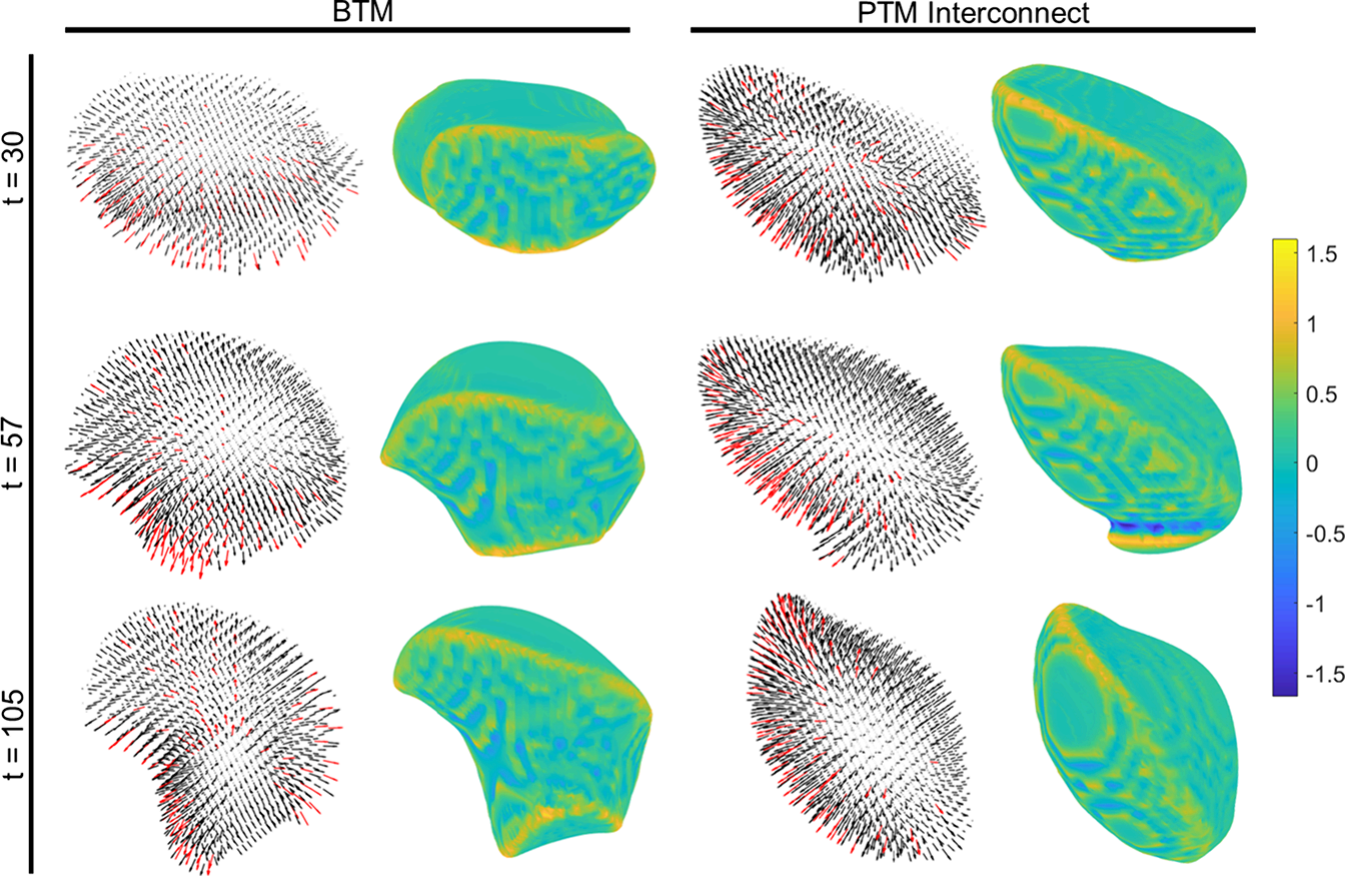
Local curvature maps, calculated as interpolations of *c* mapped to the ρ = 0.5 surface of the cell, alongside spatial maps of the *p⃗*-field vectors, for cells interacting with the (left) BTM and (right) PTM interconnect at time points at the start of the PTM interconnect interaction (*t* = 30), right after the minimum *z*_cm_ (*t* = 57), and as the cell is moving away from the interconnect (*t* = 105).

The redirection discussed here and prior, also in relation to Figure 6, offers a potential explanation for the main cell behavioral differences noted between the BTM and PTM substrates. While total histograms of membrane curvature on the BTM and PTM interconnect, particularly when the cell interrogates the interconnect itself, do not immediately demonstrate obvious or dramatic differences (Figure S8), the correlations between cell curvature, shape energy, and *p⃗*-field redirection all support the hypothesis that the substrate curvature landscape is dictating the critical elements of cell behavior in these porous materials. Further examination of the distribution of local membrane curvature and *p⃗*-field organization is of great interest in these systems, since these elements are also thought to contribute to the downstream differences in cell phenotype. Figure 9 shows curvature maps and *p⃗*-field vectors for three time points of interest in the BTM and the PTM interconnect. The curvature maps were created by taking the local curvature at any point along the membrane, which was already utilized within the existing *p⃗*-field equation as *c*. Those values were then interpolated and mapped to the ρ = 0.5 surface of the cell. These curvature maps are presented alongside the respective *p⃗*-field maps, which visualize all of the vectors in the cell (the average is taken every 3 membrane locations to improve visibility). The top 100 vectors with the highest magnitudes are displayed in red. In the curvature maps, the low curvature values appear to be associated with the contact area and top surface of each cell, whereas the highest local curvatures correspond to where the cell curves away from the substrate around its periphery. For the BTM case, the distributions of high and low curvatures are not meaningfully distinguishable from one another between the three time points. In contrast, the curvature map for the cell on the PTM interconnect resembles each other at *t* = 30 and *t* = 105, yet the map at *t* = 57, which corresponds to when the cell is interacting with the pore throat, takes on a noticeably different distribution. This instant is approximately coincident with the redirection shown in Figure 8, suggesting that the high substrate curvature around the pore throat is one of the dominating factors leading to the unique migratory behavior in PTMs.

The plotted *p⃗*-field maps in Figure 9 depict an additional unique feature of the behavior induced by the PTM interconnect. In the BTM case, all three time points show the *p⃗*-field vectors pointing outward from the cell, present somewhat randomly throughout the volume but with notable clustering near the leading edge. At *t* = 30, prior to reaching the pore throat, the PTM interconnect *p⃗*-field maps exhibit a distribution similar to that of the BTM, with high magnitude vectors at the leading edges and dispersed elsewhere throughout the cell. However, just after the cell has reached the minimum *z*_cm_ position at *t* = 57, there is a curtailing of the high-magnitude vectors at the leading edge in contact with the interconnect, altering the balance of actin polymerization between the leading and trailing edges. This imbalance becomes even more apparent after the cell has been redirected and is moving upward at *t* = 105. This phenomenon is believed to be caused, at least in part, by the inclusion of membrane tension in eq 2. Recall that this term is designed to exponentially reduce the local *p⃗*-field in regions of high curvature or dramatic change in surface area in order to penalize such configurations. Therefore, the redirection behavior seen in the PTM and the PTM interconnect is caused by a reduction in the *p⃗*-field at the high-curvature interconnect region, which then causes the net *p⃗*-field to point back into the pocket. Thus, the predominance of high curvature regions in a PTM (pore throats) and the response of the *p⃗*-field to the substrate’s curvature landscape are believed to be the main causes of the redirection and the notable differences in shape and migratory cell behavior between the BTM and PTM substrates.

The results of this model, along with existing *in vivo* data,^20^ strongly suggest a hypothesis that a porous substrate with continuous, uniform, negative Gaussian curvature is capable of modulating cell behavior and phenotype as compared to a chemically identical substrate with varying pore size and curvature, in ways that are not related to size exclusion. The work shown here supports this hypothesis and suggests that the mechanism behind this result is in part related to how substrate curvature mediates actin polymerization redirection as well as the local forces along the cell membrane (via membrane tension and local curvature). This hypothesis can be further tested via *in vitro* experiments where varying cell types, such as fibroblasts and monocyte-derived macrophages, are seeded into BTM and PTM samples. These cells can then be visualized within the porous substrates, analyzed for cell shape, and examined for functional phenotypes. Further mechanistic hypotheses could be investigated in this environment, probing the relationship between these results and pathways associated with mechanosensing, actin dynamics, and cell phenotype. Specific examples include the use of Y27632 to inhibit the ROCK signaling pathway^55,61^ or the disruption of microscale curvature sensitive septin proteins via BORG3 or forchlorfenuron^30,92−94^ to further couple the causative factors predicted by this model to *in vitro* cell behavior. Recently, the mechanically activated PIEZO1 membrane channel has also been implicated in macrophage polarization, offering an additional avenue for mechanistic investigation.^95,96^

## Conclusion

Validation of a new model on convex, dome-like substrates against *in vitro* data^79^ demonstrates the significance of membrane tension in mediating cell behavior on curved substrates and the need to include it in model development. This is particularly important when studying bijel-templated materials, given the unique curvature distribution of bijel and other spinodal structures. Additional validation by grossly replicating existing *in vitro* studies which support a relationship between macrophage cell shape and phenotype further demonstrates the potential of this model to accurately guide future *in vitro* studies examining cell behavior on curved substrates.^52^ With this model, initial studies of cell dynamics in implantable porous materials are completed. This first set of predictions, with only a basic set of cell physics involved, already suggests significant differences between actin-based BTM– and PTM–cell interactions, which begin to elucidate the mechanistic differences between induced cell behavior from these substrates. The quantification presented here suggests that these cells differ in their overall migration, shape, spatial curvature distributions, and *p⃗*-field dynamics. Compared to a PTM, a cell interacting with a BTM is more likely to migrate, have a complex cell shape, experience less curvature variation while interrogating the substrate, and have a more consistent distribution of high magnitude *p⃗*-field vectors. Additionally, our calculations of the effective shape energy reveal a highly variable energetic landscape caused by the PTM topography, which causes cells to remain at the available local energy minima. This is in contrast to the more uniform topography of the BTM, which translates to an energetic landscape that lacks preferred locations in which the cell to reside. Direct investigation of the relevance of interconnect curvature supports the hypothesis that the tendency of a cell to redirect and cease migration in a PTM is directly linked to the locally sensed curvature and membrane energy rather than the dimensions of the pores themselves. These model results are applicable to any generic cell that locomotes via actin-ratcheting, including macrophages, fibroblasts, and other cell types implicated in vascularization. Existing literature which demonstrates a connection between cell phenotype and overall cell shape or migratory pattern,^52,97^ in conjunction with these results, suggests that the induced cell shape and migration behavior on the negative Gaussian curvature of BTMs may be contributing to the observed immune benefit. Therefore, the next experimental investigation of this material should seek to determine whether this benefit is caused directly by the effect of substrate curvature on macrophage polarization via induced shape and migration. Parallel or concurrent studies could examine similar effects on other cell types, which may be influencing macrophage polarization via secondary pathways.^20^ Based on this work, future studies can also investigate how micrometer-scale membrane curvature distributions may affect mechanotransduction.

With these data, an informed approach to the *in vitro* study of cell behavior on BTMs can be developed. Further experimental validation of the predictions stated here is needed to continue improving the model for future use. Potential experiments should include specific *in vitro* curvature studies to continue bridging the gap in understanding how negative Gaussian curvature at the micron-scale impacts cell behavior and phenotype, leading to the overall improved immune response demonstrated *in vivo*.^20^ Recent investigations connecting specific membrane proteins to mechanical sensing and macrophage polarization offer a unique path to continue this work.^29,30,95,98^

## Supporting Information Available

Dimensionalization of model equations (Supporting Model Information); dimensional model parameters (Table S1); model convergence (Figure S1); additional PTM–cell interaction cases (Figure S2) with quantification (Figure S3); cell velocity (Figure S4); widened interconnect–cell interaction (Figure S5); cell–substrate contact area (Figure S6); PTM–cell interaction to *t* = 1000 with quantification (Figure S7); histogram of local membrane curvature (Figure S8) (PDF)
Video S1: Cells with (green) and without (blue) membrane tension interrogating a positive Gaussian (convex dome-like) structure (MP4)
Video S2: Two additional sets of cells with membrane tension (darker) and without membrane tension (lighter), with different initial positions, which result in the cell without membrane tension remaining atop the dome-like structure and the cell with membrane tension moving off (MP4)
Video S3: Cell motion through a BTM substrate (MP4)
Video S4: Cell motion interrogating a PTM interconnect (MP4)
Video S5: Cell motion interrogating a PTM-matching hyperboloid (MP4)
