# Supplementary material for: Effect of Porous Substrate Topographies on Cell Dynamics: A Computational Study
Source: ACS Biomater Sci Eng. 2023 Sep 15;9(10):5666–78. doi: 10.1021/acsbiomaterials.3c01008 (PMC10565724; doi:10.1021/acsbiomaterials.3c01008)
Supplement: Supplementary file 1 — ab3c01008_si_001.pdf [file ab3c01008_si_001.pdf]

Supporting Information for: Effect of Porous Substrate Topographies on Cell Dynamics: A Computational Study

Alyse R. Gonthier[1], Elliot L. Botvinick[2,3,4,5,6], Anna Grosberg*[2,3,6,7,8,9], & Ali Mohraz*[1,7]

[1]University of California, Irvine, Department of Materials Science & Engineering, CA 92697, USA
[2]University of California, Irvine, Department of Biomedical Engineering, CA 92697, USA
[3]University of California, Irvine, Center for Complex Biological Systems, CA 92697, USA
[4]University of California, Irvine, Beckman Laser Institute and Medical Clinic, CA 92697, USA
[5]University of California, Irvine, Department of Surgery, CA 92697, USA
[6]University of California, Irvine, Edwards Lifesciences Foundation Cardiovascular Innovation & Research Center, CA 92697, USA
[7]University of California, Irvine, Department of Chemical & Biomolecular Engineering, CA 92697, USA
[8]University of California, Irvine, The NSF-Simons Center for Multiscale Cell Fate Research, CA 92697, USA
[9]University of California, Irvine, Sue and Bill Gross Stem Cell Research Center, CA 92697, USA

*email addresses: grosberg@uci.edu, mohraz@uci.edu

Supporting Model Information

While the previous phase field model[61,62] was presented in a non-dimensional form, it is useful for physical understanding to start with dimensional equations, and to non-dimensionalize each term to ensure proper link to experimentally observable values for each parameter, where applicable.

Let the dimensional form of Equation 1 be:

$$\frac{\partial\tilde{\rho}}{\partial\tilde{t}} = \widetilde{D_\rho}\tilde{\nabla}^2\tilde{\rho} - (A_1 - \tilde{\rho})(\tilde{\delta} - \tilde{\rho})\tilde{\rho} - \tilde{\alpha}\tilde{\nabla}\tilde{\rho}\cdot\tilde{\vec{p}} - \tilde{\kappa}\tilde{\nabla}\tilde{\Phi}\cdot\tilde{\nabla}\tilde{\rho} - \tilde{\lambda}\tilde{\rho}\widetilde{\Phi^2} \tag{1}$$

Note, that while the phase variable ρ is inherently dimensionless, for physical understanding of the fundamental dimensions of the problem, we will track the pseudo dimensions of ρ by introducing a non-dimensionalization factor ρ_0. Similarly, $\vec{p}$ is not associated with any units, but we will track its dimensions with a factor of $\overrightarrow{p_0}$. Finally, the characteristic time is defined as t_0 and the characteristic length scale is L_0.

$$\rho = \frac{\tilde{\rho}}{\rho_0} \tag{2}$$

$$\vec{p} = \frac{\tilde{\vec{p}}}{\widetilde{\overrightarrow{p_0}}} \tag{3}$$

$$t = \frac{\tilde{t}}{t_0} \tag{4}$$

Thus,

$$\frac{\partial\rho}{\partial t} = \frac{t_0}{\rho_0}\widetilde{D_\rho}\tilde{\nabla}^2\tilde{\rho} - \frac{t_0}{\rho_0}(A_1 - \tilde{\rho})(\tilde{\delta} - \tilde{\rho})\tilde{\rho} - \frac{t_0}{\rho_0}\tilde{\alpha}\tilde{\nabla}\tilde{\rho}\cdot\tilde{\vec{p}} - \frac{t_0}{\rho_0}\tilde{\kappa}\tilde{\nabla}\tilde{\Phi}\cdot\tilde{\nabla}\tilde{\rho} - \frac{t_0}{\rho_0}\tilde{\lambda}\tilde{\rho}\widetilde{\Phi^2} \tag{5}$$

Similarly to the phase of the cell, we track the phase of the substrate, which is inherently unitless, with factors of Ψ_0 and Φ_0 to ensure consistency. The relevant dimensional gradients are defined by:

$$\tilde{\nabla}\tilde{\Phi} = \frac{\Phi_0}{L_0}\nabla\Phi \quad (6)$$

$$\tilde{\nabla}\tilde{\rho} = \frac{\rho_0}{L_0}\nabla\rho \quad (7)$$

Next, we non-dimensionalize each term:

The first term would be:

$$\frac{t_0}{\rho_0}\widetilde{D_\rho}\tilde{\nabla}^2\tilde{\rho} = \left(\frac{t_0}{\rho_0}\widetilde{D_\rho}\right)\frac{\rho_0}{L_0^2}\nabla^2\rho \quad (8)$$

and its non-dimensional counterpart therefore is:

$$D_\rho\nabla^2\rho = \left(\frac{t_0}{\rho_0}\widetilde{D_\rho}\right)\frac{\rho_0}{L_0^2}\nabla^2\rho \quad (9)$$

where the non-dimensional diffusion coefficient is defined by the characteristic time and length:

$$D_\rho = \frac{\mathrm{t}_0}{L_0^2}\widetilde{D_\rho} \quad (10)$$

The second term is more complex because of the presence of δ, so it is convenient to define temporary non-dimensional variables A_1, A_2, and A_3.

$$A_3\frac{t_0}{\rho_0}(A_1 - \tilde{\rho})(\tilde{\delta} - \tilde{\rho})\tilde{\rho} = A_3\frac{t_0}{\rho_0}(A_1 - \rho_0\rho)(\tilde{\delta} - \rho_0\rho)\rho_0\rho \quad (11)$$

$$A_3\frac{t_0}{\rho_0}(A_1 - \rho_0\rho)(\tilde{\delta} - \rho_0\rho)\rho_0\rho = A_3 t_0\rho_0^2\left(\frac{A_1}{\rho_0} - \rho\right)\left(\frac{\tilde{\delta}}{\rho_0} - \rho\right)\rho \quad (12)$$

where

$$A_1 = \rho_0 \quad (13)$$

Then evaluating δ:

$$\tilde{\delta} = \frac{1}{2}A_2 + \tilde{\mu}(\int \widetilde{\rho_t}(x,y,z)d\tilde{x}d\tilde{y}d\tilde{z} - V_i) - \tilde{\sigma}\left|\tilde{\vec{p}}\right|^2 \quad (14)$$

where

$$d\tilde{x} = L_0\, dx \quad (15)$$

such that

$$\tilde{\delta} = \frac{1}{2}A_2 + \tilde{\mu}L_0^3\rho_0(\int \rho_t(x,y,z)dxdydz - V_i) - \tilde{\sigma}\vec{p}_0^2|\vec{p}|^2 \quad (16)$$

where

$$\frac{1}{2}A_2\ [=]\ \tilde{\delta} \quad (17)$$

The three parts may therefore be treated separately, giving:

$$\mu(\int \rho_t(x,y,z)dxdydz - V_i) = \tilde{\mu}L_0^3\rho_0(\int \rho_t(x,y,z)dxdydz - V_i) \tag{18}$$

$$\mu = L_0^3\rho_0\tilde{\mu} \tag{19}$$

$$\sigma|\vec{p}|^2 = \tilde{\sigma}\vec{p}_0^2|\vec{p}|^2 \tag{20}$$

$$\sigma = \vec{p}_0^2\tilde{\sigma} \tag{21}$$

Returning to the original equation

$$A_3 t_0 \rho_0^2 \left(\frac{A_1}{\rho_0} - \rho\right)\left(\frac{\tilde{\delta}}{\rho_0} - \rho\right)\rho \tag{22}$$

We apply $\frac{1}{\rho_0}$ to the terms previously evaluated such that

$$A_2 = \rho_0 \tag{23}$$

$$\mu = L_0^3\tilde{\mu} \tag{24}$$

$$\sigma = \frac{\vec{p}_0^2}{\rho_0}\tilde{\sigma} \tag{25}$$

Thus

$$\delta = \frac{\tilde{\delta}}{\rho_0} \tag{26}$$

Finally:

$$A_3 t_0 \rho_0^2 (1-\rho)(\delta-\rho)\rho \tag{27}$$

where

$$A_3 = \frac{1}{t_0\rho_0^2} \tag{28}$$

By solving for A_1-A_3, we are now able to tie any of the parameters used in these equations to characteristic units i.e. t_0, L_0, and the characteristic units used purely for tracking of ρ_0 and Φ_0.

The third term would be:

$$\frac{t_0}{\rho_0}\tilde{\alpha}\tilde{\nabla}\tilde{\rho} \cdot \tilde{\vec{p}} = \frac{t_0}{\rho_0}\tilde{\alpha}\frac{\rho_0}{L_0}\nabla\rho(\rho_0\vec{p}) \tag{29}$$

And its non-dimensional counterpart therefore is:

$$\alpha\nabla\rho \cdot \vec{p} = \frac{t_0}{\rho_0}\tilde{\alpha}\frac{\rho_0}{L_0}\nabla\rho(\rho_0\vec{p}) \tag{30}$$

where the dimensionless coefficient is:

$$\alpha = \frac{t_0\rho_0}{L_0}\tilde{\alpha} \tag{31}$$

The fourth term would be:

$$\frac{t_0}{\rho_0}\tilde{\kappa}\tilde{\nabla}\tilde{\Phi}\cdot\tilde{\nabla}\tilde{\rho} = \frac{t_0}{\rho_0}\tilde{\kappa}\ \frac{\Phi_0}{L_0}\nabla\Phi\frac{\rho_0}{L_0}\nabla\rho \tag{32}$$

and its dimensionless counterpart is:

$$\kappa\nabla\Phi\cdot\nabla\rho = \frac{t_0}{\rho_0}\tilde{\kappa}\ \frac{\Phi_0}{L_0}\nabla\Phi\cdot\frac{\rho_0}{L_0}\nabla\rho \tag{33}$$

where the dimensionless coefficient is:

$$\kappa = \frac{t_0\Phi_0}{L_0^2}\tilde{\kappa} \tag{34}$$

The fifth term would be:

$$\frac{t_0}{\rho_0}\tilde{\lambda}\tilde{\rho}\widetilde{\Phi^2} = \frac{t_0}{\rho_0}\tilde{\lambda}\rho_0\rho\Phi_0^2\Phi^2 \tag{35}$$

and its dimensionless counterpart is:

$$\lambda\rho\Phi^2 = \frac{t_0}{\rho_0}\tilde{\lambda}\rho_0\rho\Phi_0^2\Phi^2 \tag{36}$$

where the dimensionless coefficient is:

$$\lambda = t_0\Phi_0^2\tilde{\lambda} \tag{37}$$

A similar analysis can be performed for Equation 2:

$$\frac{\partial\tilde{\vec{p}}}{\partial\tilde{t}} = \widetilde{D_{\vec{p}}}\tilde{\nabla}^2\tilde{\vec{p}} - \widetilde{\tau_1^{-1}}\tilde{\vec{p}} - \tilde{\gamma}[\tilde{\nabla}\tilde{\rho}\cdot\tilde{\vec{p}}]\tilde{\vec{p}} - \mathrm{A}_4\tilde{\Phi}^2\tilde{\vec{p}} - \tilde{\beta}\tilde{\Psi}\left[(1-\theta)\hat{P}\left(\tilde{\nabla}\tilde{\rho}\right) + \theta\tilde{\nabla}\tilde{\rho}\right] \tag{38}$$

$$\frac{\partial\vec{p}}{\partial t} = \frac{t_0}{\vec{p}_0}\widetilde{D_{\vec{p}}}\tilde{\nabla}^2\tilde{\vec{p}} - \frac{t_0}{\vec{p}_0}\widetilde{\tau_1^{-1}}\tilde{\vec{p}} - \frac{t_0}{\vec{p}_0}\tilde{\gamma}[\tilde{\nabla}\tilde{\rho}\cdot\tilde{\vec{p}}]\tilde{\vec{p}} - \frac{t_0}{\vec{p}_0}\mathrm{A}_4\tilde{\Phi}^2\tilde{\vec{p}} - \frac{t_0}{\vec{p}_0}\tilde{\beta}\tilde{\Psi}\left[(1-\theta)\hat{P}\left(\tilde{\nabla}\tilde{\rho}\right) + \theta\tilde{\nabla}\tilde{\rho}\right] \tag{39}$$

where the first term would be:

$$\frac{t_0}{\vec{p}_0}\widetilde{D_{\vec{p}}}\tilde{\nabla}^2\tilde{\vec{p}} = \frac{t_0}{\vec{p}_0}\widetilde{D_p}\frac{\overrightarrow{p_0}}{L_0^2}\nabla^2\vec{p} \tag{40}$$

and its dimensionless counterpart is:

$$D_{\vec{p}}\nabla^2\vec{p} = \frac{t_0}{\vec{p}_0}\widetilde{D_p}\frac{\overrightarrow{p_0}}{L_0^2}\nabla^2\vec{p} \tag{41}$$

resulting in the dimensionless diffusion coefficient:

$$D_{\vec{p}} = \frac{t_0}{L_0^2}\widetilde{D_p} \tag{42}$$

The second term would be:

$$\frac{t_0}{\vec{p}_0}\widetilde{\tau_1^{-1}}\tilde{\vec{p}} = \frac{t_0}{\vec{p}_0}\widetilde{\tau_1^{-1}}\vec{p}_0\vec{p} \tag{43}$$

with its dimensionless counterpart:

$$\tau_1^{-1}\vec{p} = t_0\widetilde{\tau_1^{-1}}\vec{p} \tag{44}$$

Therefore, τ_1 is the characteristic time, defined by the depolymerization of actin:

$$\tau_1^{-1} = t_0\widetilde{\tau_1^{-1}} \tag{45}$$

The third term would be:

$$\frac{t_0}{\vec{p}_0}\tilde{\gamma}[\tilde{\nabla}\tilde{\rho}\cdot\tilde{\vec{p}}]\tilde{\vec{p}} = \frac{t_0}{\vec{p}_0}\tilde{\gamma}[\frac{\rho_0}{L_0}\nabla\rho\cdot\vec{p}_0\vec{p}]\vec{p}_0\vec{p} \tag{46}$$

with its dimensionless counterpart:

$$\gamma[\nabla\rho\cdot\vec{p}]\vec{p} = t_0\tilde{\gamma}[\frac{\rho_0}{L_0}\nabla\rho\cdot\vec{p}_0\vec{p}]\vec{p} \tag{47}$$

$$\gamma[\nabla\rho\cdot\vec{p}]\vec{p} = \frac{\rho_0}{L_0}\vec{p}_0 t_0\tilde{\gamma}[\nabla\rho\cdot\vec{p}]\vec{p} \tag{48}$$

giving the dimensionless coefficient:

$$\gamma = \frac{\rho_0\vec{p}_0 t_0}{L_0}\tilde{\gamma} \tag{49}$$

The fourth term would be:

$$A_4\frac{t_0}{\vec{p}_0}\tilde{\Phi}^2\tilde{\vec{p}} = \mathrm{A}_4\frac{t_0}{\vec{p}_0}\Phi_0^2\Phi^2\vec{p}_0\vec{p} \tag{50}$$

where based on the parameters in the previous publication:

$$A_4 t_0\Phi_0^2 = 1 \tag{51}$$

Thus,

$$A_4 = \frac{1}{t_0\Phi_0^2} \tag{52}$$

The fifth term would be:

$$\frac{t_0}{\vec{p}_0}\tilde{\beta}\tilde{\Psi}\left[(1-\theta)\hat{P}\left(\tilde{\nabla}\tilde{\rho}\right) + \theta\tilde{\nabla}\tilde{\rho}\right] = \frac{t_0}{\vec{p}_0}\tilde{\beta}\Psi_0\Psi\left[(1-\theta)\hat{P}\left(\frac{\rho_0}{L_0}\nabla\rho\right) + \theta\frac{\rho_0}{L_0}\nabla\rho\right] \tag{53}$$

As $\hat{P}$ is an operator, that term will have the dimensions of $\tilde{\nabla}\tilde{\rho}$.

$$\beta\Psi\left[(1-\theta)\hat{P}(\nabla\rho) + \theta\nabla\rho\right] = \frac{\rho_0}{L_0}\frac{t_0}{\vec{p}_0}\tilde{\beta}\Psi_0\Psi\left[(1-\theta)\hat{P}(\nabla\rho) + \theta\nabla\rho\right] \tag{54}$$

resulting in the dimensionless coefficient:

$$\beta = \frac{\rho_0 t_0\Psi_0}{L_0\vec{p}_0}\tilde{\beta} \tag{55}$$

This non-dimensionalization allows us to define the characteristic units of all the parameters used in Equations 1-3, which are presented in Table S1. For consistency, the fundamental units associated with this model are defined as characteristic length (L_0), and characteristic time (t_0). For convenience of the reader, we have kept track of

the characteristic ρ_0, $\vec{p}_0$, Φ_0, Ψ_0, but please note that all of these are unitless, per the definition of the phase field model.

Dimensional Parameter	Physical Meaning	Fundamental Model Dimensions	Example Units
$\widetilde{D_\rho}$	Diffusion Coefficient of ρ	$\frac{L_0^2}{\mathrm{t}_0}$	$\frac{\mu\mathrm{m}^2}{s}$
$\tilde{\mu}$	Strength of Volume Conservation	$\frac{1}{L_0^3}$	$\frac{1}{\mu\mathrm{m}^3}$
$\tilde{\sigma}$	Permits Cell Volume Change in Response to $\vec{p}$	$\frac{\rho_0}{\vec{p}_0^2}$	Unitless
$\tilde{\alpha}$	Advection of ρ Along $\vec{p}$	$\frac{L_0}{t_0\rho_0}$	$\frac{\mu\mathrm{m}}{s}$
$\tilde{\kappa}$	Adhesion	$\frac{L_0^2}{t_0\Phi_0}$	$\frac{\mu\mathrm{m}^2}{s}$
$\tilde{\lambda}$	Cell-Substrate Exclusion	$\frac{1}{t_0\Phi_0^2}$	$\frac{1}{s}$
$\widetilde{D_p}$	Diffusion Coefficient of Actin	$\frac{L_0^2}{t_0}$	$\frac{\mu\mathrm{m}^2}{s}$
$\widetilde{\tau_1}$	Actin Depolymerization	t_0	s
$\tilde{\gamma}$	Rear-Cell Myosin Motor Contraction	$\frac{L_0}{\rho_0\vec{p}_0t_0}$	$\frac{\mu\mathrm{m}}{s}$
$\tilde{\beta}$	Actin Polymerization	$\frac{L_0\vec{p}_0}{\rho_0t_0\Psi_0}$	$\frac{\mu\mathrm{m}}{s}$

Table S1. Dimensionalized model parameters, along with their associated meanings and example units, are presented to aid in the physical understanding of this phase-field model.

Supporting Figures

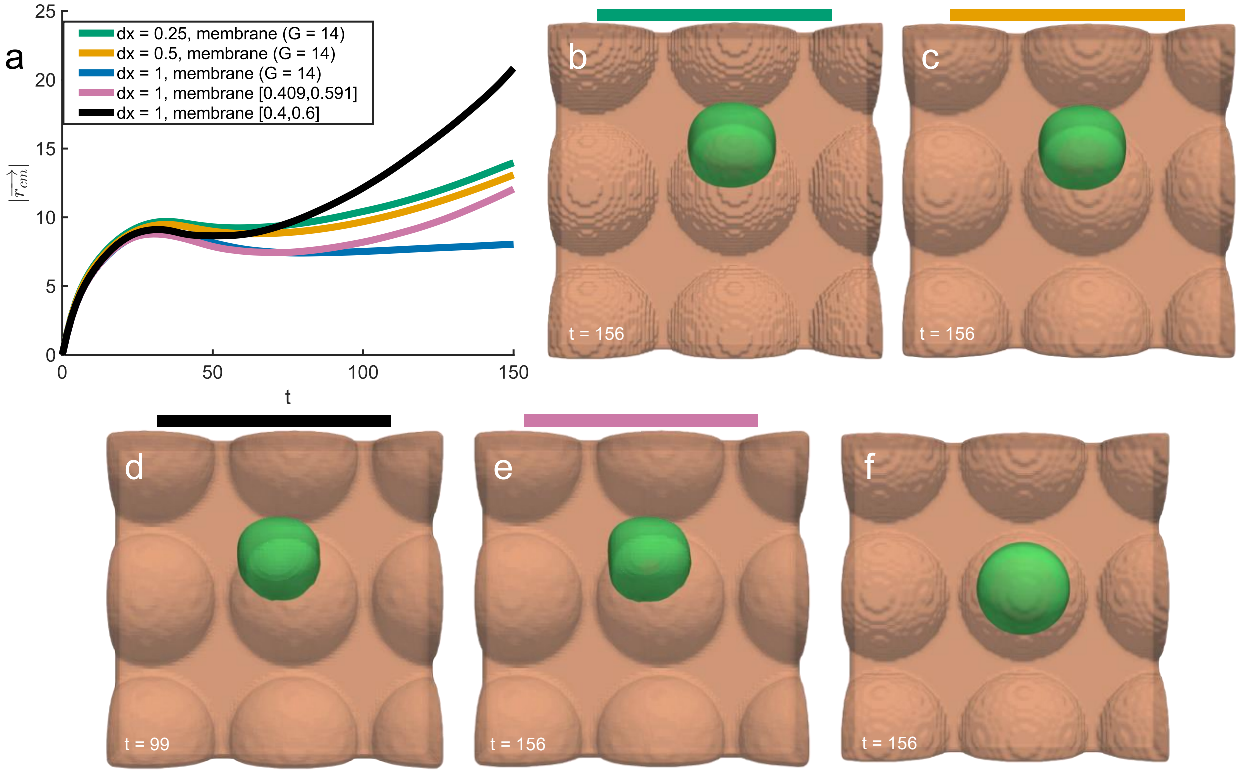

Figure S1. The results of this model are not meaningfully dependent on discretization size at dx = 0.5 and below when incorporating a continuous membrane definition: $1 - \tanh^2(G(\rho - 0.5))$. The radius from origin plot (a) demonstrates the closeness of the model predictions at (b) dx = 0.25 and (c) dx = 0.5 with a continuous membrane definition, noted by its G value. This result differs quantitatively from the dx = 1 case where the membrane is defined by a binary threshold of (d) $0.4 \leq \rho \leq 0.6$, but still matches the critical qualitative behavior seen in previous cases. The dx = 1 case where the membrane is defined by a binary threshold of (e) $0.409 \leq \rho \leq 0.591$ exhibits behavior more quantitatively similar to (b) and (c), also importantly replicating the relevant qualitative behavior. The discrepancy between (d) and (e) effectively highlights how sensitive the model is to the definition of the membrane as an arbitrary threshold of ρ, supporting the utilization of the more adaptable continuous definition usable at dx = 0.5 and below. At the coarser dx = 1, the continuous membrane definition does not reliably give appropriate weight to the membrane overall and therefore does not reproduce the qualitative behavior seen in the other cases (shown in (a)). The case at dx = 0.5 without membrane tension is included (f) as a visual reference.

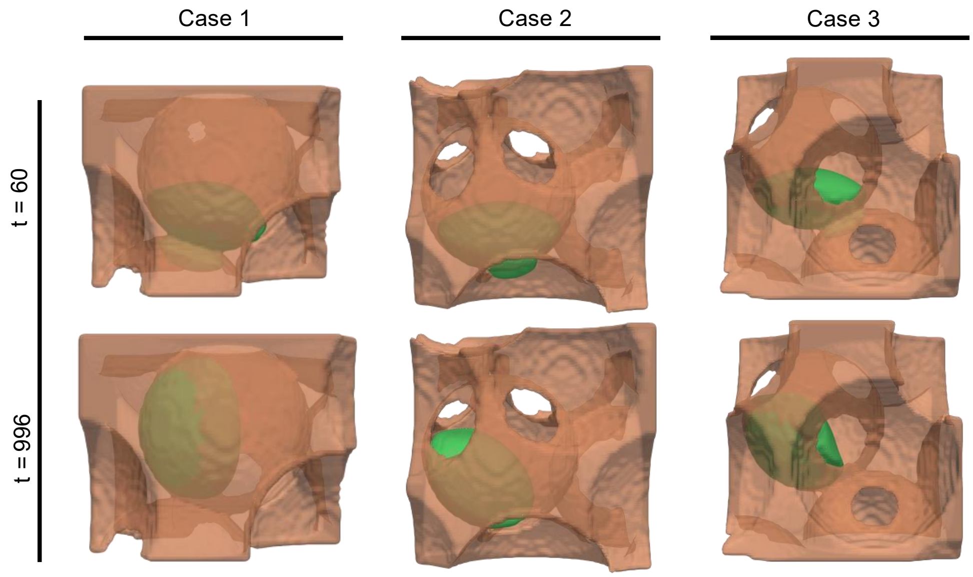

Figure S2. Three cases of cells interacting with a full PTM structure. Smaller pieces of the structure are displayed here for better visualization. Case 1 is the same as in Figure 4b, with zoomed in view. Cases 2 and 3 have all of the same initial conditions as Case 1, but are originated in different pores within a large PTM structure. In all three cases, the cell is confined to the pore in which it originates.

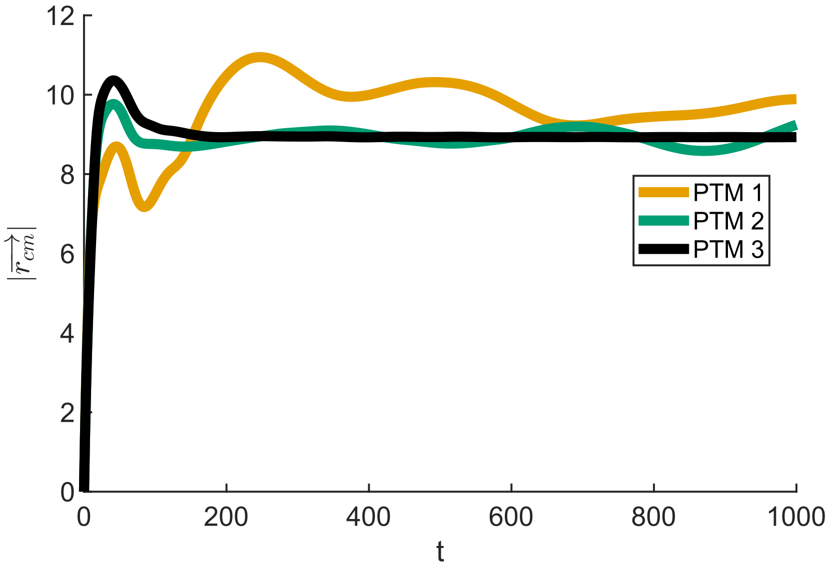

Figure S3. Radius from origin of the three PTM-interacting cells shown in Figure S2, demonstrating the lack of significant migration of each cell after initial interconnect and pore interactions occur.

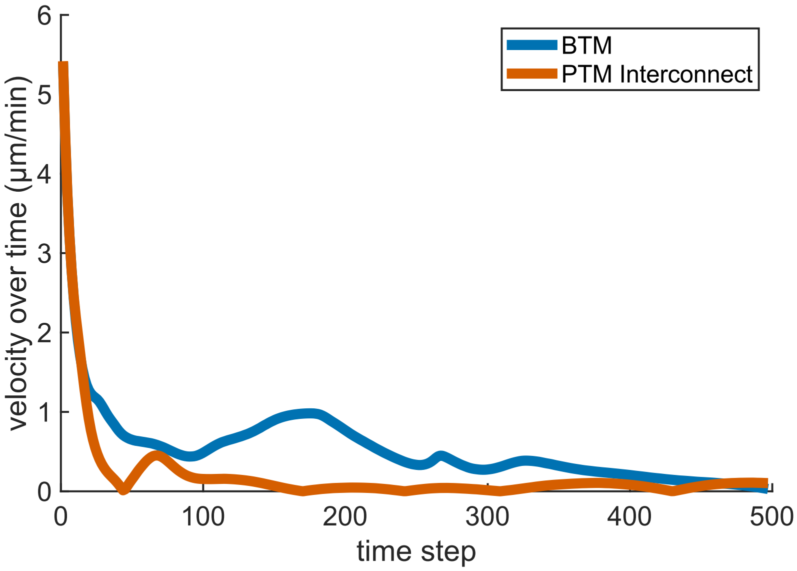

Figure S4. Speed of the cells moving through the BTM and the PTM Interconnect substrates. The range of speeds shown here once substrate interaction begins, approximately 0-2 μm/min, is within expected physiologic values for relevant cells (fibroblasts, macrophages).

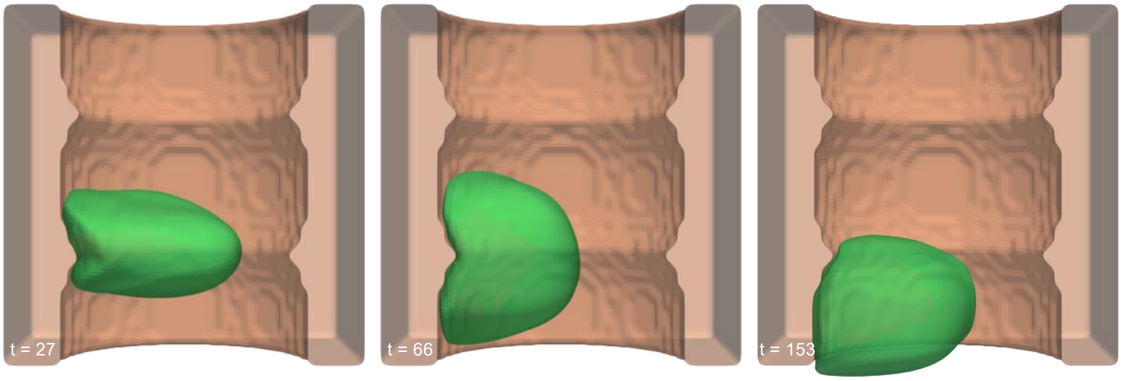

Figure S5. A large interconnect with lower local curvature at the pore throat allows a cell to traverse across it, when starting with the same initial conditions used in Figure 4. This interconnect overlap ratio (pore throat-to-pore) is over 80%, in contrast to the PTM Interconnect discussed in the main text which is based on experimentally reported substrates with an overlap ratio of approximately 30-35%.

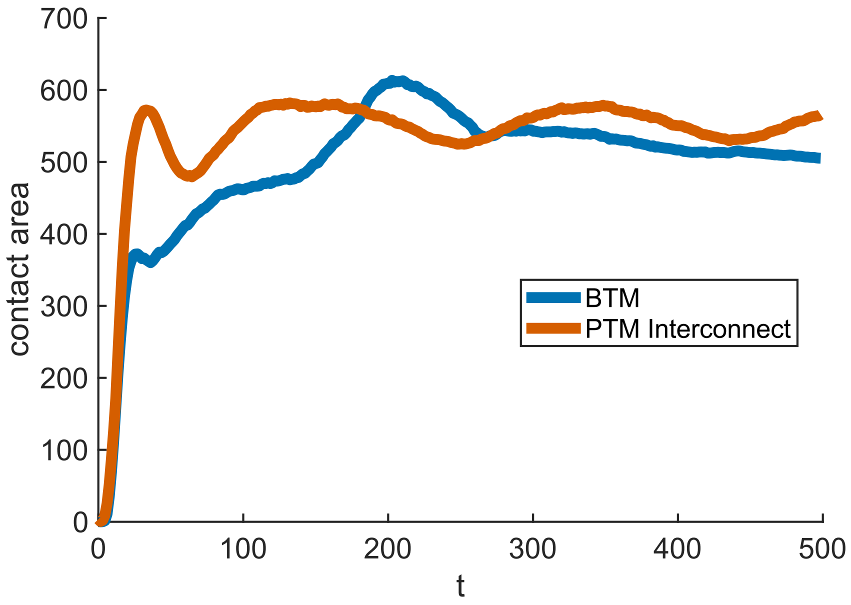

Figure S6. Contact area for cells interacting with a BTM and PTM Interconnect. Contact area is defined by the region of overlap where the cell ($\rho \geq 0.4$) and the substrate ($\Phi \geq 0.1$) coexist.

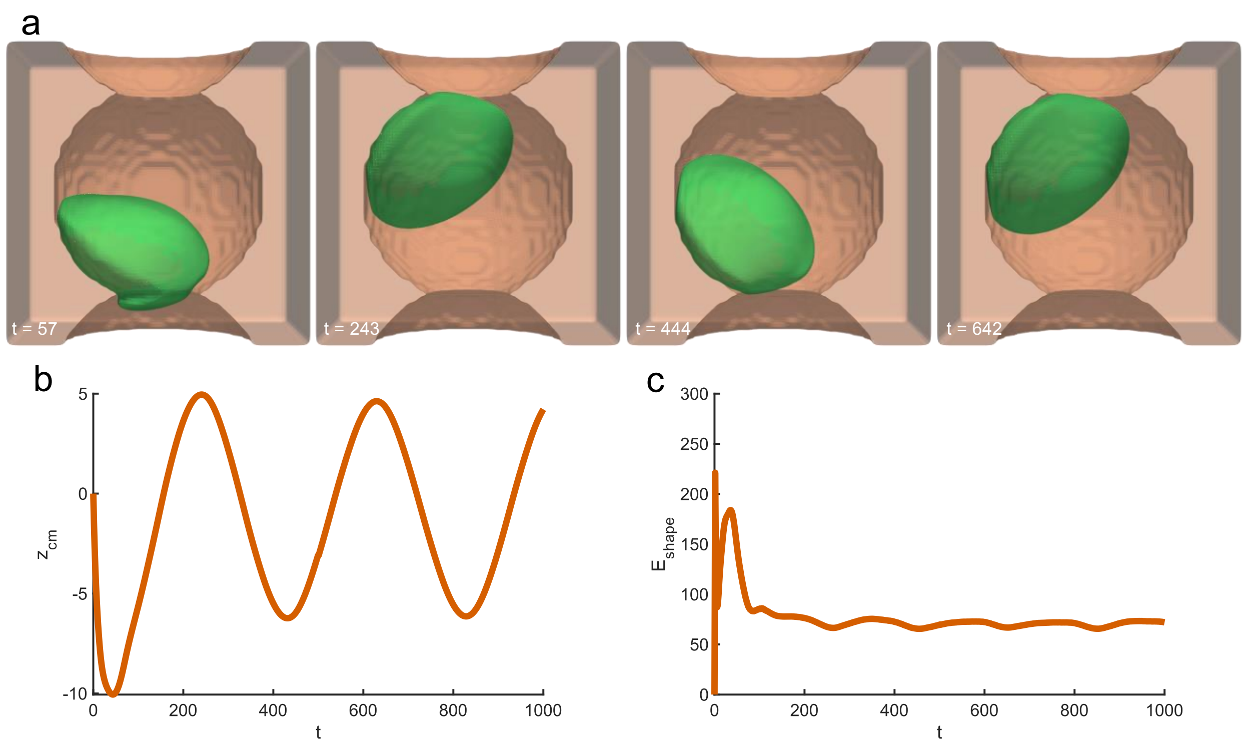

Figure S7. Demonstration of the continuous restraint of the cell within its original pore in a simplified PTM Interconnect out to t = 1000, represented visually (a), by z-position from origin (b), and shape energy (c).

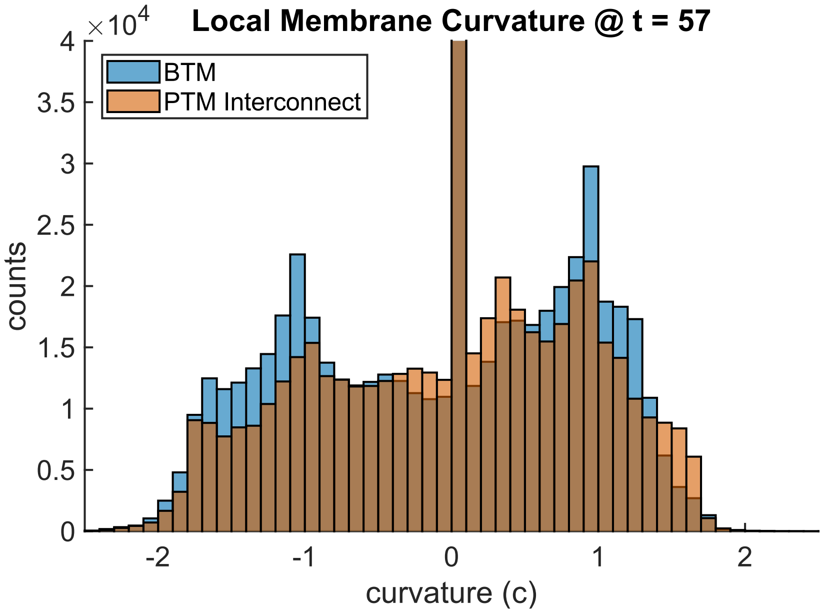

Figure S8. Histograms of the local curvatures at the cell membranes ($\chi > 0$) for a cell interacting with a BTM or PTM interconnect. For any cell interacting with a substrate, there will be some quantity of low values associated with the contact area and non-contact surfaces, as well as some quantity of high values associated with the periphery of the cell where it curves away from the substrate. We theorize that the high quantity of each of these values, which will be present in any cell, are what convolute the curvature histograms and make significant interpretation of challenging. (Note: the counts for bin 0-0.05 rise to 3.3×10^6 and 1.3×10^5 for the BTM and PTM Interconnect, respectively).

List of Supporting Videos

Video S1: Cells with (green) and without (blue) membrane tension interrogating a positive Gaussian (convex dome-like) structure. ($t_{max} = 500$)

Video S2: Two additional sets of cells with membrane tension (darker) and without membrane tension (lighter), with different initial positions, which result in the cell without membrane tension remaining atop of the dome-like structure and the cell with membrane tension moving off. ($t_{max} = 500$)

Video S3: Cell motion through a BTM substrate. ($t_{max} = 500$)

Video S4: Cell motion interrogating a PTM interconnect. ($t_{max} = 1000$)

Video S5: Cell motion interrogating a PTM-matching hyperboloid. ($t_{max} = 1000$)
